# Prevalence of Intimate Partner Violence Among Intimate Partners During the Perinatal Period: A Narrative Literature Review

**DOI:** 10.3389/fpsyt.2021.601236

**Published:** 2021-02-09

**Authors:** Amera Mojahed, Nada Alaidarous, Marie Kopp, Anneke Pogarell, Freya Thiel, Susan Garthus-Niegel

**Affiliations:** ^1^Institute and Policlinic of Occupational and Social Medicine, Medical Faculty, Technical University of Dresden, Dresden, Germany; ^2^Arthur Labatt Family School of Nursing, University of Western Ontario, London, ON, Canada; ^3^Department of Psychotherapy and Psychosomatic Medicine, University Hospital Carl Gustav Carus, Technical University of Dresden, Dresden, Germany; ^4^Department of Medicine, Faculty of Medicine, Medical School Hamburg, Hamburg, Germany; ^5^Department of Child Health and Development, Norwegian Institute of Public Health, Oslo, Norway

**Keywords:** intimate partner violence, bidirectional IPV, perinatal period, prevalence, associated factors, narrative review

## Abstract

Intimate partner violence (IPV) affects individuals and families from all backgrounds, regardless of their ethnicity, socio-economic status, sexual orientation, or religion. Pregnancy and childbirth could be a time of vulnerability to violence because of changes in physical, emotional, social, and economic demands and needs. Prevalence of IPV against women during the perinatal period is increasingly researched and documented. However, evidence on IPV prevalence among intimate partners as well as on the course of IPV over the perinatal period is scarce. The purpose of this review was to provide a narrative synthesis of the existing literature regarding the prevalence estimates of IPV among intimate partners over the perinatal period. Through this review, we also gained better insight into associated factors, as well as the various forms of IPV. Of the 766 studies assessing prevalence estimates identified, 86 were included, where 80 studies focused on unidirectional IPV (i.e., perpetrated by men against women) and six studies investigated bidirectional IPV (i.e., IPV perpetrated by both partners). Most of the included studies reported lower overall prevalence rates for unidirectional IPV postpartum (range: 2–58%) compared to pregnancy (range: 1.5–66.9%). Psychological violence was found to be the most prevalent form of violence during the entire perinatal period. Studies on bidirectional IPV mostly reported women's perpetration to be almost as high as that of their partner or even higher, yet their findings need to be interpreted with caution. In addition, our results also highlighted the associated factors of IPV among partners, in which they were assimilated into a multi-level ecological model and were analyzed through an intersectional framework. Based on our findings, IPV is found to be highly prevalent during the entire perinatal period and in populations suffering from social inequalities. Further research exploring not only the occurrence, but also the motivations and the context of the bidirectionality of IPV during the perinatal period may facilitate better understanding of the detrimental consequences on partners and their families, as well as the development of effective intervention strategies. Public health prevention approaches intervening at optimal times during the perinatal period are also needed.

## Introduction

Intimate partner violence (IPV) affects individuals and families from various ethnic, economic, religious, or sexual backgrounds. The World Health Organization (WHO) defines IPV as “any act or behavior within a present or former intimate relationship that causes physical, psychological, or sexual harm” (1). These behaviors may pertain to (1) acts of physical violence (e.g., hitting, kicking, beating); (2) sexual violence (e.g., forced sexual intercourse, sexual coercion); (3) psychological (emotional) violence (e.g., insults, humiliation, intimidation, threats of harm); (4) controlling behavior (e.g., isolation from family and friends, monitoring movements, restricting access to financial resources, employment, education, medical care) (1, 2). With approximately a third of the women worldwide having experienced IPV during their life (3), IPV represents the most common form of violence against women. The WHO multi-country study on women's health and domestic violence against women found the prevalence of physical IPV in pregnancy to range between 1% in Japan to 28% in Peru, with the majority of sites ranging between 4 and 12% (4). An analysis of Demographic and Health Surveys and the International Violence against Women Survey found prevalence rates for IPV during pregnancy between 2% in Australia, Denmark, Cambodia, and Philippines to 13.5% in Uganda, with the majority ranging between 4 and 9% (5). Clinical studies around the world, which tend to yield higher prevalence rates but often are the only sources of information available, found the highest prevalence in Egypt with 32%, followed by India (28%), Saudi Arabia (21%), and Mexico (11%) (6). A recent review of African clinical studies reported prevalence rates of 23–40% for physical, 3–27% for sexual, and 25–49% for emotional or psychological intimate partner violence during pregnancy (7). Taking into account the variations based on the cultural background and populations investigated, prevalence of IPV could be higher in specific groups, for example, those experiencing critical life events such as the transition to parenthood, which may in turn augment and intersect with already existing factors and thus increase the risk to engage in or experience IPV.

Physical health consequences of IPV perpetrated against women have great negative consequences on the mother and her offspring, including delayed prenatal care, low birth weight (LBW), intrauterine growth retardation, preterm labor, or even miscarriage (7–11). Psychological implications of IPV during the perinatal period may be of particular importance because they may also bear adverse consequences for the mother, the child and the entire family. Depression, post-traumatic stress disorder (PTSD), anxiety, panic disorders, and substance abuse disorders have been documented as the most common psychological consequences of IPV for mothers during their pregnancy and postpartum (5, 12). Maternal depression during pregnancy is associated with an increased risk for offspring's future depression (13), whereas maternal exposure to adverse life events, such as the exposure to violence during pregnancy, has been linked to offspring autism and schizophrenia (14). Maternal PTSD during pregnancy and after childbirth could impact the offspring's hypothalamo-pituitary-adrenocortical (HPA) axis regulation (15), which in turn would result in psychological disorders such as anxiety, eating disorders, and externalizing problems during childhood and later in life (16). The gravest consequence of IPV during the perinatal period is death. Several studies found that maternal injury is a leading cause of maternal mortality; 54.3% of pregnancy-associated suicides involved intimate partner conflict, whereas 45.3% of pregnancy-related femicides were associated with pre-existing IPV victimization of women (17, 18).

Despite great advances in researching IPV, little is known about how victimization experiences may be patterned over the perinatal period (i.e., during the time frame from 1 year before to 24 months after the birth of the child), and how it may represent a period of particular vulnerability to violence. Where prevalence of IPV against women alone is increasingly researched and documented during the perinatal period, reported evidence on bidirectional IPV (i.e., perpetrated by both partners) prevalence is still scarce. Women's IPV perpetration has detrimental health consequences on both partners (19). It increases men's and women's risk for substance abuse and depression (20). While the context of violence toward men has been proven to be very different for women in that it represents defensive or retaliatory behavior, violence common to both partners can nonetheless result in a more stressful and dangerous living environment for children (21). In fact, IPV among intimate partners is associated with child maltreatment and reduced social-emotional child development (22–25). Therefore, it appears to be imperative to not only investigate prevalence estimates of IPV perpetrated against women alone, but to also improve our understanding of bidirectional IPV during pregnancy and postpartum in order to inform the ongoing process of developing effective screening and interventions for women and their families. The purpose of this review is to provide a narrative synthesis of the existing literature regarding the prevalence estimates of IPV among partners over the perinatal period as well as any associated factors. These factors will be analyzed through an intersectional approach that considers individual, family, community, and societal related factors within an ecological model.

## Materials and Methods

### Search Strategy

A systematic search of the available literature was performed in March 2020 from the following databases: PubMed, Embase via Ovid, CINAHL, and Scopus. The search strategy was developed according to the PICO model to determine search concepts and types of studies. The keywords (and their combinations) adopted for the research are the following: perinatal, perinatal women, perinatal men, perinatal couple, intimate partner violence, IPV, domestic violence, spousal abuse, prevalence, observational studies. Separate searches for each primary database combined Medical Subject Subheadings (MeSH) terms and key text words with the Boolean operators (AND) and (OR), accordingly. The full list of search terms for PubMed can be found in Appendix A.

### Eligibility Criteria

All publications in English, German, and Arabic languages that appeared between 2000 and 2020 have been considered. For studies to be included in this review, the search was international and had to include a sample that refers to IPV victims affected by it during the perinatal period (i.e., the time frame from 1 year before to 24 months after the birth of the child). The target population were intimate partners, regardless of the nature of their intimate relationship. Only empirical quantitative studies such as cohort, case-control, and cross-sectional studies were included. Qualitative studies were excluded. We considered IPV the primary outcome for this review.

### Data Collection Process

**Figure 1:**
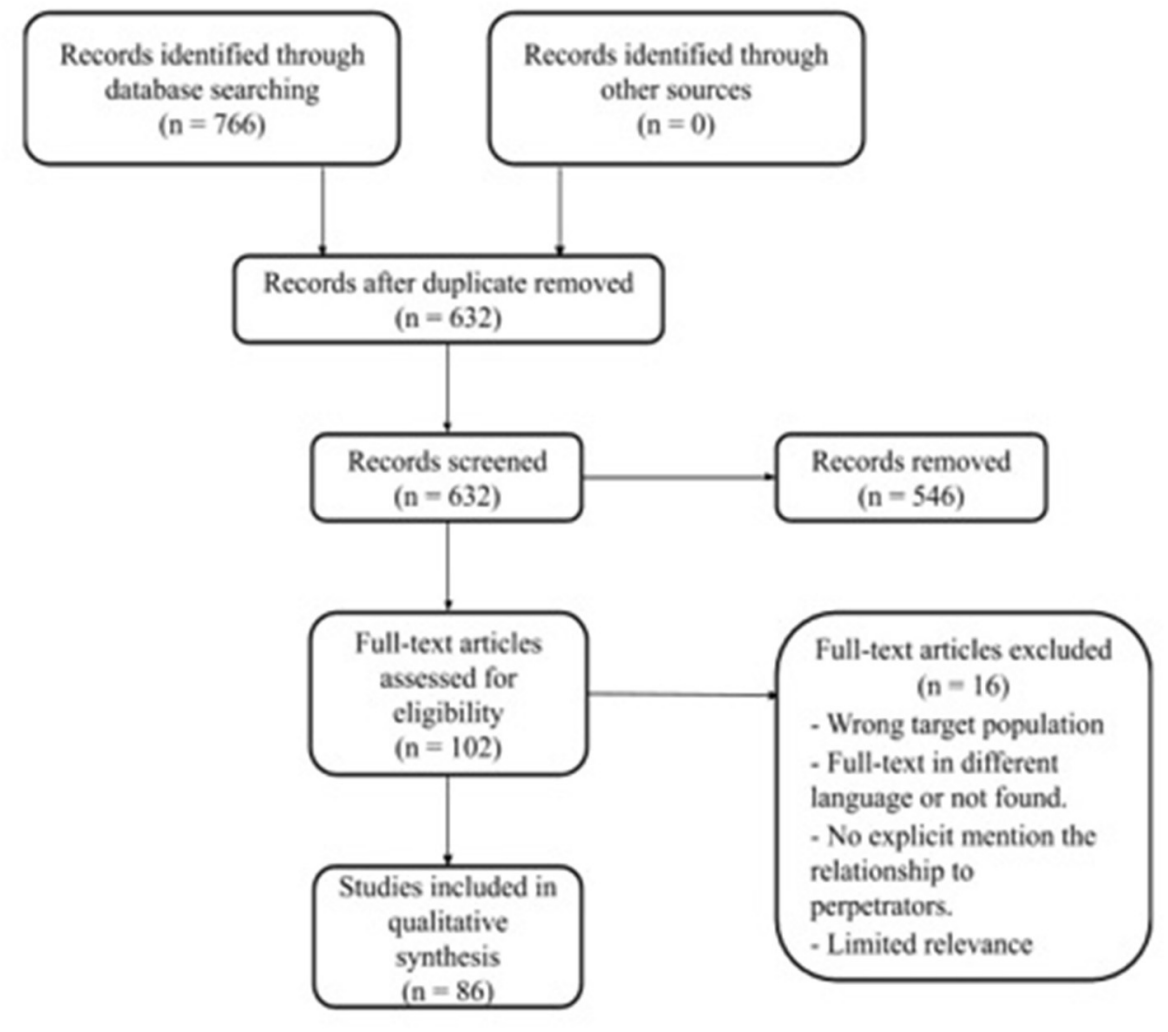
PRISMA flow chart.

A flowchart of the search and inclusion process is presented in Figure 1. The search provided a total of 766 articles. After removing duplicates, a total of 632 papers were collected and imported into a web-based tool, Rayyan QCRI (26). The abstracts of these articles were checked, in which 546 abstracts demonstrated no relevance for this review and were excluded. Assessment of eligibility of the 102 full-text articles lead to exclusion of 16 articles because they did not report the relationship to perpetrators (i.e., being an intimate partner or a natal family member, etc.), nor did they provide any prevalence estimates. The remaining 86 studies will be described in the results section.

### Data Synthesis

A qualitative approach was employed in synthesizing the results. Since prevalence studies of IPV tend to be highly heterogeneous and violence definitions tend to vary among research settings, we did not consider conducting any quantitative analyses for this review. The relevant data were tabulated in a data extraction form that was developed. Prevalence estimates of IPV among intimate partners, as well as associated factors relevant for IPV during the perinatal period were constructed. For each paper, we extracted and systematized the following information: author and year of publication; setting (e.g., clinical- or population-based); study design; sample size (e.g., final sample, response rate); the directionality of IPV (i.e., uni- or bidirectional); overall IPV prevalence estimates (i.e., during pregnancy, postpartum, or both); and its types (i.e., physical, sexual, psychological, economic). In addition, we also considered population characteristics and associated factors significant to IPV prevalence when available, using a multi-level ecological model where each factor is assimilated into the following levels: (a) the individual level, which represents the biolo- gical and personal history of the individuals; (b) family level, which represents factors relating to the immediate context where abuse took place; (c) community level, which represents factors relating to the formal or informal social institutions or structures in which violent relationships are embedded; and (d) societal level, which represents factors relating to gender inequality, religious or cultural belief systems, societal norms, and economic or social policies (10, 27).

## Results

### Study Selections

An overview of the study selection process can be found in Figure 1. Eighty-six studies met the inclusion criteria (28–113). The majority of the studies were cross-sectional (*n* = 75) and few used longitudinal designs (*n* = 11). The studies originated from 35 countries, published in English, and recruited only women (*n* = 90,895) (Appendix B). Eighty of the included studies investigated violence against women where the perpetrator was their current or former intimate partner. Six studies explored bidirectional perpetration of IPV, in which women can be both perpetrators as well as victims. Three terms were used to describe the violence, i.e., IPV, Gender-Based Violence (GBV), and Domestic Violence (DV). We excluded studies that reported perpetrators other than intimate partners, such as family members, since the aim of the present review was to summarize and describe the prevalence of violence perpetrated by intimate partners, as well as to investigate what factors were associated with the prevalence of IPV during the perinatal period.

### Prevalence Estimates of Unidirectional IPV and Its Types

According to the results from the included studies, we found that IPV prevalence estimates were reported either during pregnancy (*n* = 60) or during the postpartum period (*n* = 5). Further, some studies reported comparable estimates during both pregnancy and the postpartum period (*n* = 9), whereas others reported estimates during the entire perinatal period (*n* = 2) (Table 1).

**Table 1:** Prevalence estimates of unidirectional IPV during the perinatal period.

**Perinatal period**	**Country**	**Overall IPV**	**Physical**	**Psychological**	**Sexual**	**Economic**	**Study ID**
			**violence**	**violence**	**violence**	**violence**	
**During pregnancy**							
	USA	-	19%	-	-	-	Alhusen et al. (29)
		8.9%	-	-	-	-	Koenig et al. (78)
		14.5%	-	-	0.9%	-	Lutgendorf et al. (89)
	Portugal	43.4%	21.9%	43.2%	19.6%	-	Almeida et al. (30)
	Turkey	11.1%	-	-	-	-	Arslantaş et al. (32)
		31.7%	8.1%	26.7%	9.7%	-	Karaoglu et al. (73)
	Nigeria	34.4%	50.9%	68.5%	-	-	Ashimi & Amole (33)
		32.5%	27.5%	5.9%	9.8%	-	Ezeanochie et al. (47)
		-	8.1%	51.7%	1.7%	-	Ezechi et al. (48)
		17.7%	10.8%	66.2%	2.7%	-	Fawole et al. (50)
		12.6%	26.5%	1.4%	10.7%	-	Gyuse et al. (61)
		7.8%	11.2%	43.5%	1.8%	6.8%	Jeremiah et al. (71)
		44.6%	-	60.1%	-	-	Onoh et al. (94)
		-	10.3%	-	-	-	Umoh et al. (108)
	Brazil	-	6.5%	19.1%	-	-	Audi et al. (114)
		-	4.6%	-	-	-	Fiorotti et al. (55)
		34.6%	-	-	-	-	Massumi Okada et al. (86)
	Ethiopia	41.1%	21%	29.1%	19.8%	-	Azene et al. (34)
		58.7%	32.2%	57.8%	7.6%	-	Fekadu et al. (51)
		-	44.2%	39.1%	23.7%	-	Yohannes et al. (112)
	India	-	7.1%	30.6%	10.4%	-	Babu et al. (35)
		29.7%	26.9%	79.1%	33.2%	37%	Garg et al. (58)
		12.3%	10%	10.7%	1.8%	-	Jain et al. (67)
		-	13%	-	-	-	Peedicayil et al. (97)
	South Africa	21%	15%	15%	2%	-	Bernstein et al. (37)
		15%	76%	81%	26%	-	Field et al. (52)
		-	29%	32%	20%	-	Malan et al. (84)
		41%	17%	26%	5%		Modiba et al. (87)
	Mexico	18.6%	10.8%	5.9%	4%	-	Cervantes-Sanchez et al. (40)
		43.8%	15.8%	72.9%	11.3%	-	Romero-Gutierrez et al. (99)
	Jordan	15.4%	-	-	-	-	Clark et al. (42)
		40.9%	34.7%	28.1%	15.5%	-	Okour & Badarneh (92)
		-	10.4%	23.4%	5.7%	-	Oweis et al. (96)
	Uganda	26.7%	60.6%	59.6%	39.4%	-	Clarke et al. (43)
		57%	-	-	-	-	Kaye et al. (75)
		27.8%	10.6%	22.2%	10%	-	Epuitai et al. (46)
	Iran	55.9%	10.2%	43.5%	17.3%	-	Farrokh-Eslamlou et al. (49)
	Sweden	1.5%	0.4%	1%	0.1%	-	Finnbogadóttir et al. (53)
		2%	0.7%	1.6%	0.1%	-	Finnbogadóttir et al. (54)
	Israel	5.4%	20.3%	21.6%	4.1%	-	Fisher et al. (56)
	Pakistan	35%	Minor: 27% Severe: 6%	-	-	-	Habib et al. (62)
		38%	14%	24%	14%	-	Karmaliani et al. (74)
		5.7%	-	-	-	-	Sohail & Qadir (105)
	Malaysia	35%	12.9%	29.8%	9.8%	-	Haron et al. (63)
		35.9%	12.9%	29.8%	9.8%	-	Khaironisak et al. (76)
	Bangladesh	66.4%	35.2%	65%	18.5%	-	Islam et al. (67)
	England	17%	14.7%	14.3%	-	-	Johnson et al. (72)
	Japan	15.9%	-	-	-	-	Kita et al. (77)
	Belgium, Iceland, Denmark, Estonia, Norway, and Sweden	-	2.2%	2.7%	0.4	-	Lukasse et al. (80)
	Kenya	37%	10%	29%	12%	-	Makayoto et al. (83)
		66.9%	29.9%	55.8%	39.2%	-	Owaka et al. (95)
	Sri Lanka	15.9%	-	-	-	-	Muzrif et al. (90)
	Vietnam	35.2%	32.2%	3.5%	10%	-	Nguyen et al. (91)
	Jamaica	41%	-	-	-	-	Pitter & Dunn (98)
	Zimbabwe	63.1%	15.9%	-	38%	-	Shamu et al. (102)
	Nepal	27.2%	3%	16.6%	17.3%	-	Sherstha et al. (103)
	Tanzania	-	19%	-	-	-	Stöckl et al. (107)
	Nicaragua	32%	13%	32%	7%	-	Valladares et al. (109)
	Belgium	10.6%	0.5%	-	10.1%	-	Van Parys et al. (110)
	Taiwan	-	6.9%	-	-	-	Yang et al. (111)
**Postpartum**							
Within 2 years	India	37%	31%	28%	6%	-	Ahmed et al. (28)
Within 1 year	Iran	58%	21%	54%	21%	-	Amiri et al. (31)
	Sweden	2%	-	-	-	-	Rubertson et al. (100)
At 3 months	USA	21.3%	-	-	-	-	Harrykissoon et al. (64)
At 6 months		16%					
At 12 months		17.7%					
At 18 months		17.7%					
At 24 months		12.8%					
During 48 h after delivery	Iran	-	25%	35%	-	-	Salari & Nakhaee (101)
**During pregnancy and postpartum**							
During pregnancy	Nepal	26.2%	9.4%	15%	16.1%	-	Bhatta & Assanangkornchai (38)
6–10 weeks postpartum		20%	4.8%	15.2%	7.3%	-	
During pregnancy and 6 weeks postpartum	India	15%	12%	8%	2%	-	Das et al. (44)
During pregnancy and postpartum		28.4%	-	-	-	-	Silverman et al. (104)
During pregnancy and postpartum (3–6–12 months)	Australia	17%	2.2%	9%	-	-	Gartland et al. (59)
During pregnancy	South Africa	21.3%	8.7%	16.6%	3%	-	Groves et al. (60)
Postpartum (first 9 months)		17.7%	-	-	-	-	
During pregnancy	Bangladesh	66.4%	35%	18.5%	18.5%	-	Islam et al. (66)
Postpartum (first 6 months)		63.6%	32.2%	60.8%	15.5%	-	
During pregnancy and postpartum	Iran	60.6%	14.6%	60.5%	23.5%	-	Jahanfan & Malekzadegan (68)
During pregnancy		56%	-	-	-	-	Jamshidimanesh et al. (70)
Postpartum		-	5%	51.3%	-	-	
During pregnancy		42%	10%	33%	17.3%	-	Mohammadhosseini et al. (89)
Postpartum (6 to 18 months)		53.5%	14.7%	42.7%	25%	-	
During pregnancy	Tanzania	-	12.4%	31%	9%	48.4%	Mahenge et al. (82)
Postpartum (first 9 months)		-	5.2%	17.8%	3.8%	11.4%	
During pregnancy	Brazil	3.7%	14%	32.9%	0.1%	-	Marcacine et al. (85)
Postpartum		25.6%	4.3%	25.1%	0.5%	-	
During pregnancy	Nigeria	28%	-	-	-	-	Olagbuji et al. (93)
Postpartum (first 6 weeks)		0.8%	-	-	-	-	
During perinatal period	England	-	9.6%	24%	-	-	Kothari et al. (79)
	Ghana	46%	17%	34%	15%	-	Spangenberg et al. (106)

The overall IPV prevalence during pregnancy ranged from 1.5 to 66.9%, being highest in Kenya (96) and lowest in Sweden (54). During pregnancy, prevalence of psychological violence was the most prevalent form of violence and ranged from 1% in Sweden (54) to 81% in South Africa (53), followed by physical violence, ranging from 0.4% in Sweden (54) to 60.6% in Uganda (44). Sexual violence was reported in 40 studies, with a range between 0.1 and 39.4%. Prevalence estimates for economic violence were reported in two studies only: in Nigeria with 6.8% (72) and in India with 37% (59).

Moreover, the overall IPV prevalence during the 1st year postpartum ranged from 2% in Sweden (102) to 58% in Iran (32). One study reported prevalence estimates within 2 years postpartum (28) for overall IPV (37%) as well as other forms of violence i.e., physical violence (31%), psychological violence (28%), and sexual violence (6%). One study reported estimates of IPV at 3, 6, 12, 18, and 24 months postpartum, with the overall IPV prevalence rate being highest at the earliest measurement point after birth, i.e., 3 months postpartum (21,3, 16, 17.7, 17.7, 12.8%, respectively) (66). In Iran, a study reported IPV prevalence estimates for physical (25%) and psychological violence (35%) during the first 48 h after delivery.

Studies reporting prevalence estimates of IPV both during pregnancy as well as at follow-ups during the postpartum period provided comparable estimates before and after childbirth. A study from Nepal reported a decrease of prevalence rates from pregnancy to 6–10 weeks postpartum for overall (26.2–20%), physical (9.4–4.8%), and sexual IPV (16–7.3%) with an exception for psychological violence, where the prevalence rate remained the same (15%) (40). A study from Bangladesh reported a slight decrease in prevalence estimates for overall (66.4–63.6%), physical (35–32.2%), and sexual IPV (18.5–15.5%) during the first 6 months postpartum compared to the time during pregnancy. However, psychological violence was reported to have significantly increased from 18.5 to 60.8% during the first 6 months postpartum compared to the time during pregnancy (68). In Iran, a study reported increased prevalence estimates for overall (42–53.3%), physical (10–14.7%), psychological (33–42.7%), and sexual IPV (17.3–25%) during 6–18 months postpartum compared to the time during pregnancy (91).

A South African study also reported a decrease in prevalence rates, where overall IPV decreased from 21.3–17.7% during the first 9 months postpartum compared to the time during pregnancy. Prevalence estimates for physical (8.7%), psychological (16.6%), and sexual violence (3%) only occurred during pregnancy (62). Furthermore, a study from Tanzania reported higher prevalence rates during pregnancy for physical (12.4%, 5.2%), psychological (31%, 17.8%), sexual (9%, 3.8%), and economic violence (48.4%, 11.4%) compared to the first 9 months postpartum (84). Prevalence rates for overall (3.7–25.6%) and sexual IPV (0.1–0.5%) were reported to increase postpartum in comparison to the time during pregnancy, whereas physical (14%, 4.3%) and psychological violence (32.9%, 25%) seemed to be higher during pregnancy (87). In Nigeria, a study reported a 20% decrease in overall IPV prevalence (0.8%) during the first 6 weeks postpartum compared to the time during pregnancy (20.8%). Further four studies reported prevalence estimates of IPV during pregnancy and postpartum without providing any comparable estimates before and after childbirth (46, 61, 70, 106) (Table 1).

Lastly, prevalence estimates during the entire perinatal period, where no differentiation between before and after childbirth was made, were reported in two studies. A study in England found only psychological and physical violence to be prevalent, with 24 and 9.6%, respectively (81). Another study from Ghana reported psychological violence as most prevalent with 34%, followed by 17% for physical violence, and 15% for sexual violence (108).

### Prevalence Estimates of Bidirectional IPV and Its Types

Only six studies investigated bidirectionality of IPV. In the studies focused on bidirectional IPV, two of these studies were during pregnancy (37, 89), one study was during the postpartum period (46), and three studies were during both pregnancy and the postpartum period (42, 58, 66) (Table 2).

**Table 2:** Prevalence estimates of bidirectional IPV during the perinatal period.

**Perinatal period**	**Country**	**Study ID**	**Setting & sample size**	**Prevalence of IPV Victimization**	**Prevalence of IPV Perpetration**	**Remarks**
During pregnancy	Iran	Mohammad-Alizadeh-Charandabi et al. (88)	Clinical-based: public health care centers/posts in Tabriz, Iran Sample: 408 pregnant women (first 6-months)	Overall: Adolescents: 69.1% Adults: 69.8%	Overall: Adolescents: 72.1% Adults: 71%	Population: 136 adolescents (15–19) and 272 adults (19–29) Reported lifetime IPV linked to perpetration by pregnant women
		Bahrami-Vazir et al. (36)	Clinical-based: public health care centers/posts in Tabriz, Iran Sample: 525 pregnant women (24–30 weeks)	Psychological: 58% Physical: 22% Sexual coercion: 30%	Psychological: 65% Physical: 19% Sexual coercion: 15%	No data on incidents of IPV victimization prior perpetration by pregnant women
During postpartum	Brazil	Moraes et al. (45)	Clinical-based: two-stage cluster sampling from 27 primary care clinics (pediatrics) in the city of Rio de Janeiro Sample: mothers of infants up to 6 months (6-months PP)	Overall: 18.3% Minor physical: 17.5% Severe physical: 7.9%	Overall: 25% Minor physical: 23.2% Severe physical: 11.2%	Reported data on reciprocity of violence within couple
During pregnancy and postpartum	United States	Charles & Perreira (41)	Clinical-based: stratified random sample of hospital births in 20 large US cities Baseline: 4,898 pregnant women Follow-up: 3,830 (1-year PP)	Overall during pregnancy: 8.5% Overall during postpartum (1-year): 30%	Overall during pregnancy: 13.4% Overall during postpartum (1-year): 34%	
		Flanagan et al. (57)	Clinical-based: two university-affiliated health clinics Baseline: 180 pregnant women Follow-up: 122 (6-weeks PP)	Overall during pregnancy: 11.7% Overall during postpartum (6-weeks): 9.4%	Overall during pregnancy: 9.4% Overall during postpartum (6-weeks): 7.4%	
		Hellmuth et al. (65)	Clinical-based: two university affiliated health clinics between Baseline: 132 pregnant women Follow-up: 73 (6-weeks PP)	Overall during pregnancy: 67.7% Overall during postpartum (6-weeks): 54.1%	Overall during pregnancy: 72.2% Overall during postpartum (6-weeks): 64.8%	Reported data on IPV perpetration by women without history of victimization.

Few studies presented the prevalence of IPV victimization and perpetration during the perinatal period over time (Table 2). At baseline (i.e., during pregnancy), a range between 8.5 and 67.7% of women endorsed at least one instance of IPV victimization and 9.4–72.2% endorsed at least one instance of IPV perpetration. At follow-up (i.e., during postpartum), a range between 12.3 and 54.1% of women endorsed at least one instance of IPV victimization and 7.4–64.8% endorsed at least one instance of IPV perpetration. After childbirth, two studies suggest that prevalence of IPV perpetration declined for about 10% (58, 66), whereas Charles & Perreira (41) reported around 20% increase in prevalence (42). In regard to IPV victimization, only Hellmuth et al. (66) reported around 10% increase in prevalence estimates, while others suggest a decrease in prevalence rates for <20% (42, 58). In addition, it was noted that although there is a percentage of women endorsed perpetrating some form of violence against their intimate partners during the perinatal period, it was not clear if this violence was reciprocal or not. Only one longitudinal study (66) reported no reciprocity of IPV perpetration endorsed by women (i.e., 12% during pregnancy and 7% during postpartum). Reciprocity of violence within couples was defined as the endorsement of both perpetration of violence against their partner and victimization of violence by their partner (Table 3) (46).

**Table 3:** Prevalence of types of bidirectional IPV during the perinatal period at baseline and follow-up.

**Study ID**	**Sample size**	**Baseline**			**Follow-up**		
		**Type of IPV**	**Victimization**	**Perpetration**	**Type of IPV**	**Victimization**	**Perpetration**
Charles & Perreira, (41)	Baseline: 4,898 pregnant women Follow-up: 3,830 (1-year PP)	- Physical - Emotional	Overall: 8.5% 1.7% 7.5%	Overall: 13.4% 8.2% 7.0%	- Physical - Emotional - Sexual coercion	Overall: 30% 3.1% 17.3% 21.4%	Overall: 34% - 13.3% 27.7%
Flanagan et al. (57)	Baseline: 180 pregnant women Follow-up: 122 (6-weeks PP)	- Sexual only - Sexual with psychological or physical	Overall: 11.7% 1.7% 10.0%	Overall: 9.4% 9.4%	- Sexual	Overall: 12.3% 1.6% 10.7%	Overall: 7.4% 0.8% 6.6%
Hellmuth et al. (65)	Baseline: 132 pregnant women Follow-up: 73 (6-weeks PP)	- Psychological - Severe physical - At least one type	Overall: 67.7% 13.3% 8.3% Not reported.	Overall: 72.2% 21.1% 9.4% 12%	- Psychological - Severe physical - At least one type	Overall: 54.1% 10.7% 4.1% Not reported	Overall: 64.8% 20.5% 12.3% 7%

There is a limited consistency in reporting the prevalence of types of IPV victimization or perpetration across the perinatal period. For example, Bahrami-Vazir and colleagues (45) investigated the prevalence of subcategories of IPV perpetration during pregnancy, such as psychological (58%), sexual (30%), or physical violence (22%) (37). Similarly, Charles & Perreira (42) reported only the prevalence rates of physical violence (1.7%) and emotional violence (7.5%) experienced by pregnant women. They also reported prevalence rates of subcategories of IPV during postpartum, such as physical (3.1%) and emotional violence (17.3%), as well as controlling behavior (21.4%). Other authors categorized IPV types based on severity. In Hellmuth et al.'s (65), women who participated during pregnancy reported experiences of severe physical violence (8.3%) and minor psychological violence (13.3%) (66), while another study found that women during postpartum endorsed victimization of minor physical violence (17.5%) and severe physical violence (7.9%) (46). Mohammad-Alizadeh-Charandabi et al. (88) compared prevalence of IPV between age groups, i.e., adolescents (15–19 years of age) and young adults (20–29 years of age) (89). They found that, during pregnancy, sexual IPV victimization was significantly more common in both adolescents and adults, conversely, psychological IPV perpetration was significantly more common than victimization only among the adolescents.

#### Associated Factors Related to Unidirectional IPV During the Perinatal Period

In the following, we focus on associated factors found to be significantly related to IPV either during pregnancy or during the postpartum period. Other studies reported factors during both pregnancy and the postpartum period, whereas even others reported estimates during the entire perinatal period.

**In pregnancy**, 45 studies investigated associated factors of IPV (Table 4).

**Table 4:** Factors associated with unidirectional IPV.

**Perinatal period**	**Ecological model**	**Associated factors**	**Risk factor**	**Protective factor**	**Study ID**
During pregnancy	Individual level (victim-related)	Lower education	X		(30, 32, 34, 36, 43, 51, 54, 63, 95, 98, 112, 113)
		Younger age	X		(35, 36, 51, 53, 64, 72, 76, 92)
		Unemployment	X		(52, 53, 72, 98)
		Being self-employed	X		(62)
		Marital status	X		(30, 38, 53, 64)
		Mental health issues	X		(34, 38)
		Alcohol use	X		(38)
		Drug use	X		(64, 77)
		Having previous experience of IPV	X		(92, 103, 110)
		Having witnessed or been a victim of physical violence during childhood	X		(34, 43, 54, 55, 64, 76, 77, 98, 100)
		Inappropriate utilization of prenatal care services for pregnant women	X		(40)
		Early initiation of antenatal care		X	(35)
		Dowry demand	X		(67, 98)
		Low ability for decision-making, low self-esteem	X		(68, 97)
	Individual level (perpetrator-related)	Younger age	X		(34, 78, 96, 103, 104)
		Lower education	X		(35, 50, 57, 97, 104)
		Drug use	X		(34–36, 43, 44, 51, 52, 68, 77, 78, 96, 98, 103, 104, 112, 113)
		Unemployment	X		(34, 50, 57, 59, 72, 112)
		Having witnessed or been a victim of physical violence during childhood	X		(100)
	Family level	Partner's control of woman's reproductive health	X		(44, 103, 108)
		Having previous abortions	X		(78)
		Multigravidity	X		(56, 70, 93)
		Multi- and low parity	X		(36, 68, 72, 77, 78, 93, 98, 109)
		Financial distress/insufficient income	X		(53–55, 57, 100)
		Women as sole providers	X		(34)
		Husband's jealousy	X		(98)
		Polygamous marriages	X		(33, 76, 95)
		Undesired pregnancy	X		(53, 54, 93, 97)
		Pressure to have a male child	X		(70, 93)
		Unwanted marriage	X		(32)
	Community level	Being related more distantly		X	(43)
		Less frequent communication with her natal family	X		(43)
		Rural residency	X		(35, 68, 91)
		Lack of social support	X		(92, 98, 104)
		Urban residency	X	X	(30, 36, 63, 93)
	Societal level	Ethnicity (i.e., jewish or non-caucasian)	X		(30, 33, 57)
		Immigrant status	X		(30)
		HIV-positive	X		(48, 49)
		Having HIV-positive child	X		(48)
		Religion (e.g. Catholic, Muslim)	X		(56, 59, 91)
		High degree of religiosity	X		(57)
		Having supporting attitudes toward violence	X		(43, 52, 77, 104)
Postpartum	Individual level (victim-related)	Younger mothers	X		(28, 31, 102)
		Institutional delivery		X	(28)
	Individual level (perpetrator-related)	Sexual dissatisfaction	X		(31)
	Family level	Unplanned pregnancy	X		(31, 102)
		Giving birth to female child	X		(31)
		Having more than one child	X		(102)
During pregnancy and postpartum	Individual level (victim-related)	History of IPV	X		(39, 61, 90)
		Lower education	X		(90)
		Regular alcohol use during pregnancy and puerperium	X		(94)
		Employment	X		(45)
	Individual level (perpetrator-related)	Alcohol use	X		(45)
	Family level	Longer duration of marriage	X		(39)
		Insufficient income	X		(45, 60, 90)
	Community level	Controlling behavior of mother in-law	X		(39)
	Societal level	Belonging to an ethnic minority (i.e., Janajati)	X		(39, 61, 90)
		HIV-positive	X		(94)

At ***the individual level***, risk factors were either related to victims or perpetrators of IPV. Victim-related factors such as pregnant women's lower education (30, 32, 34, 36, 43, 51, 54, 63, 95, 98, 112, 113), younger age (35, 36, 51, 53, 64, 72, 76, 92), unemployment (52, 53, 72, 98), or being self-employed (62), marital status (30, 38, 53, 64), mental health issues (34, 38), alcohol use (38), drug use (64, 77), having previous experience of IPV (92, 103, 110), and having witnessed or been a victim of physical violence during childhood (34, 43, 54, 55, 64, 76, 77, 98, 100) were all associated with higher victimization of IPV. Inappropriate utilization of prenatal care services for pregnant women (40) constituted another significant association, whereas early initiation of antenatal care could be considered a protective factor (35). Moreover, dowry demand (67, 98), low ability for decision-making as well as low self-esteem (68, 97) were also associated with increased risk for IPV. ***Perpetrator-related factors***related to IPV included perpetrator's younger age (34, 78, 96, 103, 104), lower education (35, 50, 57, 97, 104), substance use, including alcohol (34–36, 43, 44, 51, 52, 68, 77, 78, 96, 98, 103, 104, 112, 113), unemployment (34, 50, 57, 59, 72, 112), and having witnessed or been a victim of physical violence during childhood (100).

At the ***family level***, factors such as those relating to marriage, family life, conflict within the family, family's living conditions are explored and included at this level. Partner's control of woman's reproductive health (103) like husband's prohibition of contraception use (44, 108), having previous abortion experience (78), multigravidity (56, 70, 93), multiparity (36, 68, 77, 78, 93, 98, 109), and low parity (72) were significantly associated with increased IPV victimization for women. Financial factors were explored in six studies. IPV increased when the family had financial distress/insufficient income (53–55, 57, 100), or when the women were the providers and the ones responsible for the family's income (34). Further factors like accusations of extramarital affair by husbands (98) or polygamous marriages (33, 76, 95) were explored and found to be statistically significant. A number of studies found the risk of violence increased by undesired pregnancy (53, 54, 93, 97), the pressure on pregnant women to have a male child (70, 93), and by being forced into marriage (32). In contrast, results of Azene et al. (34) indicated that women choosing their husband on their own, i.e., without relying on their family, is associated with IPV in pregnancy (35).

At the ***community level***, factors relating to the extended family, family's residency, and the nature of marriage are explored and included. Pregnant women being related to their husbands more distantly, as well as their less frequent communication with their natal family (43) were found to be a significant factor for increasing IPV. Living in rural areas (35, 68) such as tea plantation sectors in Sri Lanka (91), and lack of social support (92, 98, 104) were found to increase the odds of experiencing IPV. On the contrary, urban residency (36, 63, 93) was also linked to IPV. However, in another study, urban residency was found to be a protective factor against IPV (30), see Table 4.

At the ***societal level***, factors relating to the cultural context are heavily influenced by the social, religious, and political systems and should be included at this level. Pregnant women with a certain ethnicity such as being Jewish women of Sephardic descent, (57), being non-Caucasian (30, 33), with an immigrant status (30), being HIV-positive (48, 49) and having an HIV-positive child (48), or belonging to a certain religion, i.e., Catholic, Muslim, or Hindu (56, 59, 91), as well as endorsing a higher degree of religiosity (religious vs. non-religious) were at higher risk for IPV (57). Studies found that women who endorsed violence supporting attitude were also at risk for experiencing IPV (43, 52, 77, 104).

**During the postpartum period**, three studies investigated associated factors of IPV (28, 31, 102).

At the ***individual level***and as victim-related factors, IPV risk was significantly higher for younger mothers and those *unable* to fully meet the sexual expectations of their husbands (31). Institutional delivery opposed to home birth was found to be a protective factor against IPV (28).

At the ***family level***, unplanned pregnancy (31, 102), husband being disappointed about infant gender (i.e., having female children) (31), and having more than one child (102) were significantly related to IPV, see Table 2.

**During both pregnancy and the postpartum period**, six studies investigated associated factors with IPV (39, 45, 60, 61, 90, 94). Victim-related factors at the ***individual level*** included history of IPV (39, 61, 90), women who have lower education (90), and women reporting regular alcohol use during pregnancy and puerperium (94). One study reported higher risk of IPV for employed women (45). As for perpetrator-related factors, one study reported husband's alcohol use (45).

At the ***family level***, longer duration of marriage (39), and insufficient income (45, 60, 90) constituted risk factors (see Table 2).

At the ***community level***, controlling behavior of the mother-in-law was associated with higher victimization of IPV (39). At the ***societal level***, belonging to an ethnic minority (e.g., Janajati ethnicity in Nepal) (39, 61, 90) and being HIV-positive (94) were found to be associated with increased IPV victimization.

#### Associated Factors Related to Bidirectional IPV During the Perinatal Period

Among the studies examining bidirectional perpetration, four of them investigated associated factors of IPV (Table 5).

**Table 5:** Factors associated with bidirectional IPV.

**Perinatal period**	**Ecological model**	**Associated factors**	**Risk factor**	**Protective factor**	**Study ID**
During pregnancy	Individual level	Partner's dissatisfaction with their employment status	X		(36)
Postpartum	Individual level	Insufficient prenatal and postpartum medical care	X		(45)
		Younger age	X		
		Lower education	X		
		Insecure employment status	X		
	Family level	Unwanted pregnancy	X		
		Not living with the partner	X		
		Living in a household with more than one child younger than 5 years of age	X		
	Societal level	Ethnicity (i.e., African)	X		
During pregnancy and postpartum	Individual level	Lower education	X		(41)
		Substance use	X		(65)
		Alcohol abuse	X		(65)
		Being separated from child's father		X	(41)
		Stress and depression	X		(65)
	Family level	Lower dyadic adjustment	X		(65)
	Societal level	Ethnicity (i.e., Hispanic)	X		(41)

**In pregnancy** and at the ***individual level***, intimate partners' dissatisfaction with their own employment status constituted an associated variable for bidirectional IPV during pregnancy (37).

**During the postpartum period** and at the ***individual level***, insufficient prenatal and postpartum medical care, lower education and/or insecure employment status of mothers were reported to be associated factors (46).

At the ***family level***, unwanted pregnancy was found to be associated with bidirectional IPV, as well as not living with a partner, or living in a household with more than one child younger than 5 years of age (46).

At the ***societal level***, Moaes et al. (45) also reported that black adolescent mothers were at higher risk to experience IPV.

**During both pregnancy and the postpartum period** and at the ***individual level***, maternal stress due to unwanted pregnancy and feeling unsafe in one's neighborhood, lower education status, partner's substance use was associated with higher prevalence rates of bidirectional IPV. Also, IPV during pregnancy was a strong predictor of violence after childbirth, especially in constellations where both partners perpetrated violence against each other reciprocally (42). Hellmuth et al. (65) reported associated factors for reciprocal IPV, such as reported alcohol abuse in partners as well as stress and depression.

At the ***family level***, family structure was strongly associated with interpersonal violence, i.e., women who were single or uninvolved with their previous partner at the time of their child's birth were four times more likely to have been involved in a violent relationship during pregnancy (42). Lower dyadic adjustment (i.e., a process with consequences that can be identified with the rate of a couple's problematic conflicts, interpersonal tensions, individual anxiety, marital satisfaction, coherence, integrity, and collaboration about important problems) (115) was found to be an associated factor (66).

At the ***societal level***, Hispanic and other mothers in relation to white mothers were more likely to experience or perpetrate violence and abuse during pregnancy (42).

## Discussion

Our review aimed at examining prevalence estimates of IPV victimization and perpetration over the perinatal period. Moreover, we were interested in associated factors as well as the various forms of IPV during this period.

### Prevalence of Unidirectional IPV and Its Types

The narrative synthesis of relevant data revealed that most of the included studies reported on IPV during pregnancy with overall prevalence rates ranging from 1.5 to 66.9%. Less research concentrated on IPV during the postpartum period. Here, overall prevalence estimates ranged from 2 to 58%. The considerable variation of prevalence estimates found is indicative of considerable between-study variation. Hence, included studies were conducted in heterogeneous countries and investigated diverse populations with different cultural backgrounds and gender role distributions among women and men. Also, definitions of IPV, methods, and time of measurement differed markedly. Gazmararian et al. (113) already pointed out that such factors may affect prevalence estimates of IPV in pregnancy (116). Therefore, our results indicate that between-study variation could be of influence across the entire perinatal period.

Of special interest are studies reporting prevalence estimates during both pregnancy and the postpartum period. Here, the course of IPV over the perinatal period could be examined. Most of the included studies reported lower overall IPV prevalence rates postpartum compared to pregnancy. At first glance, this finding seems counterintuitive, as pregnancy clearly does not prevent the occurrence of intimate partner violence, regardless of its many negative health implications for women and their unborn child. Our findings add to the conflicting evidence of whether intimate partner violence increases or decreases during pregnancy (117). However, factors associated with IPV in this period ought to be considered when trying to explain this finding. In fact, a study found that prevalence estimates of IPV during pregnancy could be higher because expectant mothers may think staying with the violent partner is the safer option for their unborn child. Lost energy, low self-esteem, and hoping that the violence ends after the pregnancy constitute further possible reasons (54). Various forms of IPV were found including psychological, physical, sexual, and economic violence. Here, again prevalence rates, as well as types of IPV under investigation differed markedly across studies. Psychological violence was found to be the most prevalent form. This is consistent with previous research (7). The included studies focused primarily on psychological, physical, and sexual violence, while economic violence had been investigated by two studies only. This however could disregard the consequences of this type of violence and its relevant inclusion within the definition of IPV. As economic violence is often used as a controlling mechanism as part of a larger pattern of intimate partner violence (118). Despite the broad consensus that IPV, by definitions, includes all forms of sexual violence (119), an Iranian study (71) stated clearly the exclusion of questions on sexual violence and marital rape from their investigations due to cultural reasons (p. 8). This is an indication that sexual violence might be under researched in some contexts and prevalence rates could be even higher in reality (27).

### Prevalence of Bidirectional IPV and Its Types

Despite the clear research focus on unidirectional IPV, six of the included studies investigated bidirectional IPV among partners in pregnancy and/or postpartum. However, these data were solely based on women's reports. The results of these studies show the prevalence of IPV perpetration of women to be almost as high as or even higher than their victimization both during and after pregnancy. This is similar to the findings based on the two path-breaking national family violence surveys conducted by Straus & Gelles (119) which suggest gender symmetry of IPV, indicating that women are as likely to perpetrate violence as men. However, it is argued that women tend to overestimate their violence against their partners (120). This could be attributed to “their likelihood to remember their own aggression because it is deemed less appropriate and less acceptable for women than for men and thus takes on the more memorable quality of a forbidden act or one that is out of character” [(121): p. 405]. In addition to overestimating their own violence, women may also tend to underestimate their partner's violence given the norms of domestic life, which frequently find women discounting, downplaying, or normalizing their partner's violent behavior (120). Furthermore, these studies reported missing information regarding the context of the violence perpetrated by women. This could be due to the instrument used in most of the bidirectional studies (i.e., CTS-2), which has been assumed to be framing the occurrence of violence within the context of conflict resolution, which is of crucial importance in international settings where multiple populations are under examination at once (122). Most importantly, CTS-2 provides limited information about the context, initiation pattern, severity, intention, and motivation of abuse that many researchers consider central features of IPV (122). Research has consistently indicated that women's IPV perpetration is motivated mostly in self-, or in their children's defense, rather than driven by control and/or punishing motives (120, 123). Therefore, further enhanced research needs to be done to not only identify the occurrence, but also the context of the violence perpetrated by women during the perinatal period, in order to improve our understanding of the implications of this violence on their partners and their families.

### Associated Factors

Risk factors for IPV during the perinatal period may often be similar to risk factors for IPV in general. Still, given that pregnancy and the postpartum period are times that may demand increased relationship commitment and the resources needed, shedding more light on some risk factors are likely to be important here. Our narrative review revealed that most of the risk factors relating to unidirectional IPV were detected in studies focusing on IPV during pregnancy. Victim- and perpetrator-related factors at the individual level constituted both younger age and lower socioeconomic status, as well as having experienced or witnessed physical violence during childhood. This is found to be consistent with previous research (4, 7, 27). For the victimized pregnant women alone, early initiation of antenatal care (ANC) was found to be a protective factor for IPV. This could be attributed to the early detection and intervention of IPV, which possibly prevented further victimization (124). The same could be said for women who give birth in clinical settings vs. women who give birth at home, where their IPV victimization is found to decrease postpartum. Associated factors such as alcohol and drug use, insufficient utilization of prenatal care services, and reduced ability in decision-making as well as low self-esteem were also found to increase the risk of being victimized. However, previous research shows that such factors would rather be considered as consequences, where a multitude of pregnancy-specific health behaviors, as well as damaged self-image are common implications of IPV (125). Furthermore, a study reported that partners' sexual dissatisfaction could place mothers at higher risk for IPV postpartum. This could be attributed to the fact that the women are not as sexually available as their partners would like them to be, especially during this period. The patriarchal structure of some cultural contexts, which endorse the idea that a woman should be ready to satisfy her partner's sexual desires under any circumstances and at any cost could explain the higher risk for IPV victimization. This may suggest that the more patriarchal the societies the more such factors might play a role in the occurrence of IPV (27, 126). Family level related factors consisted of unplanned and undesired pregnancies, having multiple abortions, multigravidity, as well as having more (or fewer) than two children. As previous research pointed out, such factors could be considered as consequences of IPV, where some would be attributed to the partner's control over the woman's reproductive health or injury caused by assaultive episodes (27, 125, 127). Of relevant associated factors to IPV were the pressure on women to have a male child, which increased women's risk for victimization during pregnancy, as well as partners' disappointment with the child's gender (i.e., being female), which contributed to increased risk for victimization postpartum. These findings are consistent with previous evidence (27). Associated factors with bidirectional IPV were found to be similar to those regarding unidirectional IPV. Of special interest, women who perpetrated violence had partners with poorer dyadic adjustment, greater depression and stress levels, as well as greater severity of reported alcohol abuse compared to women who did not perpetrate IPV. Although causal attributions cannot be made here, further research is warranted to identify detrimental outcomes that are key indicators of mental, emotional, and physical health.

### Applying an Intersectional Approach

The studies included have traditionally identified individual characteristics and features of the social context that may be important for understanding violence against women. This scope of analysis often overlooks the power dynamic and impact of overlapping identities that are shaping the living realities of individuals and pushing them to the margins of society. An intersectional approach analyzes these identities, which could help enhance our understanding of how they coexist and shape individuals' lives in the community. Here, the findings reveal the interrelatedness of the factors mentioned thus far with the factors at the societal level like ethnicity (e.g., Jewish, African, or Hispanic women), having immigrant status, being HIV-positive, or having an HIV-positive child) indicate that the intersectionality lens is of essential importance in the context of our review. Instead of viewing characteristics such as age, socioeconomic status, class, gender, or race individually or as parts of an individual (128), an intersectional perspective views the influence of these characteristics as a process within a structural context of overlapping and interlocking identities. Such factors therefore appear not only to predispose pregnant women and mothers to IPV but it may worsen pre-existing violence. For example, as an immigrant woman, in addition to being confronted with gender inequalities, she is also faced with structural violence (i.e., injustices embedded in economic, political and cultural structures) of the host society (30). Consequently, IPV is a more complex problem for immigrant women and has serious consequences based on their social identities. As a person with a Jewish, African, or Hispanic racial identity, she faces racial discrimination (racism). As a woman, she faces sexism, which includes gender inequality, prejudice, stereotyping, or discrimination based on gender. Another form of discrimination would be social classism, which is discrimination based on a person's economic position in society that is determined mainly by income, educational attainment, financial security, and other criteria. Race is proven to influence social class standing. Likewise, gender and class are related because women continue to be underrepresented in high-level and highly paid positions but overrepresented in low-paying jobs (129, 130). Her multiple interlocked identities of race, gender, and class determine her lived experiences of violence. This implies that power relations intersect to produce specific vulnerabilities for specific groups in specific contexts. Moreover, new insights on the intersecting inequities resulting from different systems of domination (e.g., racism, sexism, classism), and varying forms of discrimination at community and societal levels (e.g., medical care, education, or employment) can help in highlighting the need for tailored prevention and intervention strategies for IPV (131).

## Strengths and Limitations

Strengths of this review lie in the systematic search for relevant literature, the systematic process of data extraction, and its focus on prevalence estimates of IPV and its varying forms among partners, as well as their associated factors. Nonetheless, some limitations ought to be considered. Due to the narrative design of the review, no meta-analyses of the reported IPV prevalence estimates were conducted. Therefore, no pooled estimates were presented. Our hypothesis that the considerable variation of prevalence estimates found is attributable to between-study variation was not tested.

## Conclusions

This work contributes to the literature by providing prevalence estimates of IPV among intimate partners as well as its associated factors during the perinatal period. Higher prevalence estimates were reported during pregnancy, with an overall IPV prevalence ranging from 1.5 to 66.9%, followed by an overall IPV prevalence of 2–58% during the postpartum period. Psychological violence was found to be the most prevalent form during the entire perinatal period compared to physical or sexual violence. Our results also highlighted the relationship between IPV and the varying associated factors, which relate to the different levels of the ecological model, suggesting a complex pattern of intersecting factors, which could put pregnant and/or postpartum women or partners at greater risk for IPV victimization. Studies regarding bidirectional perpetration of IPV during the perinatal period have been explored, yet their findings need to be interpreted with caution. Further research exploring not only the occurrence, but also the motivations and the contexts of the bidirectionality of IPV during the perinatal period may facilitate better understanding of the detrimental consequences on partners and their families, as well as better understanding of the detrimental consequences on partners and their families, as well as the development of effective intervention strategies. Public health prevention approaches intervening at optimal times during the perinatal period, are also needed. As a future outlook, as part of the recently started INVITE study (study on **IN**timate partner **VI**olence **T**reatment pr**E**ferences), our research group will generate a more comprehensive view of intervention preferences and barriers reported by postpartum women, who could be exposed to IPV and/or suffer from mental health problems.

## Author Contributions

AM, NA, MK, and SG-N designed and conceptualized the present study. AM and NA developed the search strategies. AM, NA, MK, AP, and FT conducted manuscript screening, data extraction, and wrote the first draft of the manuscript. SG-N supervised data extraction and drafting of the manuscript. AM, NA, MK, and SG-N contributed to the manuscript revision. All authors read and approved the submitted version.

## Conflict of Interest

The authors declare that the research was conducted in the absence of any commercial or financial relationships that could be construed as a potential conflict of interest.
