# Supplementary material for: Prevalence of Intimate Partner Violence Among Intimate Partners During the Perinatal Period: A Narrative Literature Review
Source: Front Psychiatry. 2021 Feb 9;12:601236. doi: 10.3389/fpsyt.2021.601236 (PMC7900188; doi:10.3389/fpsyt.2021.601236)
Supplement: Supplementary file 1 [file Table_1.DOCX]

***Supplementary Material***

# **Appendix A. Search Strategy**

| **PubMed** | **P** | #1 (perinatal[Title/Abstract]) AND pregnant[Title/Abstract] |
| --- | --- | --- |
|  | **I** | #2 ((((((((((((((((((((((intimate partner violence[Title/Abstract]) OR IPV[Title/Abstract]) OR intimate partner abuse[Title/Abstract]) OR intimate violence[Title/Abstract]) OR partner violence[Title/Abstract]) OR partner abuse[Title/Abstract]) OR domestic violence[Title/Abstract]) OR domestic abuse[Title/Abstract]) OR spouse abuse[Title/Abstract]) OR spousal abuse[Title/Abstract]) OR spousal violence[Title/Abstract]) OR family violence[Title/Abstract]) OR couple violence[Title/Abstract]) OR marital violence[Title/Abstract]) OR marital abuse[Title/Abstract]) OR physical violence[Title/Abstract]) OR physical abuse[Title/Abstract]) OR sexual violence[Title/Abstract]) OR sexual abuse[Title/Abstract]) OR emotional violence[Title/Abstract]) OR emotional abuse[Title/Abstract]) OR psychological violence[Title/Abstract]) OR psychological abuse[Title/Abstract] |
|  | **O** | #3 (prevalence[Title/Abstract]) OR prevalences[Title/Abstract] |
|  | **S** | #4 ((((((((longitudinal[Text Word]) OR prospective[Text Word]) OR cohort studies[MeSH Terms]) OR cohort study[MeSH Terms]) OR retrospective[Text Word]) OR cases[Text Word]) OR controls[Text Word]) OR cross sectional[Text Word]) |
|  |  | #1 AND #2 AND #3 AND #4 -> 17 |

#

**Appendix B. Extraction table for included studies**

| **Study ID -**  **Country** | | **Setting**  **Study design**  **Sample size (response rate)** | **Directionality of IPV** | **Prevalence of IPV & its types** | **Perinatal period** | **Population's characteristics and associated factors** | **Significant associations with IPV** |
| --- | --- | --- | --- | --- | --- | --- | --- |
| 1 | Ahmad et al., 2016  India | population-based  cross-sectional  4223 | unidirectional | Overall GBV (last 12 months): 37%  Emotional: 31%  Physical: 28%  Sexual: 6%  Perpetrator(s):  husbands | During postpartum (within 2 years PP) | Most women aged 15 to 49 years having  a child below 2 years  All women were living with their husbands for the last 6 to12 months  Only 14% women were working in addition to household work; 59% had no formal education | Associated with:  incorrect reproductive health behaviors, pregnancy complications,  poor birth preparedness, poor likelihood of institutional delivery, limited  postnatal care, and limited spousal communication for family planning.  After controlling for socio-economic variables in multivariate analysis, only pregnancy complications |
| 2 | Alhusen et al., 2013  USA | population-based  prospective, longitudinal  166 | unidirectional | Physical: 19%  Perpetrator(s):  husbands | During pregnancy (gestational week 24-28) | Most women were African American, at least 16 years of age, spoke English  as their primary language.  About 44% were married/partnered; 23% employed at least part-time; 66% less than high school diploma. | Associated with:  marijuana use; increase in having a Small for Gestational Age neonate. |
| 3 | Almeida et al., 2017  Portugal | population-based  cross-sectional  852 | unidirectional | Overall IPV: 43.4%  Physical: 21.9%  Psychological: 43.2%  Sexual: 19.6%  Perpetrator(s):  husbands | During pregnancy | Most women were hospitalised in the maternity ward; Caucasian; over 18; married; received education up to year 9 | Associated with:  being immigrant; non-Caucasian; single/divorced/widowed; academic qualifications; monthly income<1000 euros  Through logistic regression, the following protective factors have been identified: nationality (Portuguese), race (Caucasian) and place of residence (city). |
| 4 | Amiri et al., 2018  Iran | population-based  cross-sectional  398 | unidirectional | Overall IPV: 58%  Psychological: 54%  Physical: 21%  Sexual: 21%  Perpetrator(s):  husbands | During postpartum (12 months) | Most women were married and living with the husband during the past year;  15% employed; a third less than high school diploma | Associated with:  age less than 30 years; unplanned pregnancy; husband disappointment about their baby’s gender; inability to fully meet the husband’s sexual expectations. |
| 5 | Arslantaş et al., 2012  Turkey | population-based  cross-sectional  253 | unidirectional | Overall DV: 11.1%  Perpetrator(s):  husbands | During pregnancy | Most women were married; over 15 years old; received education | Associated with:  primary school or lower level of education and unwanted marriage |
| 6 | Ashimi & Amole, 2015  Nigeria | population-based  cross-sectional  326 | unidirectional | Overall DV: 34.4%  Physical: 50.9%  Verbal: 68.5%  Perpetrator(s):  current husband in 37%; co-wives in 31%; in-laws in 23%; siblings in 9.3%; former husband/  partner in 4.7% | During pregnancy (median GW 28) | Most women were married (57% monogamous; 43% polygamous); between 16-45 years old; 36% unemployed; a third less than high school diploma | Associated with:  ethnicity and type of marriage were identified as predictors for DV |
| 7 | Audi et al., 2008  Brazil | population-based  cross-sectional  1379 | unidirectional | Psychological:19.1%  Physical/sexual:5%  Perpetrator(s):  husbands | During pregnancy | Most women were  married or stable union; 25% working; received junior high education | Associated with:  adolescent intimate partner and the pregnant woman had witnessed physical aggression before she was 15 years old; difficulties in attending antenatal appointments; intimate partner uses drugs and does not work; low level of education of the pregnant women; the pregnant woman being responsible for the family; pregnant woman had suffered physical aggression during childhood; presence of common mental disorder; and intimate partner consumes alcoholic beverage twice or more weekly |
| 8 | Azene et al., 2019  Ethiopia | institution-based  cross-sectional  409 | unidirectional | overall IPV: 41.1%  psychological: 29.1%;  physical: 21%; sexual  violence: 19.8%  Perpetrator(s):  husbands | During pregnancy | Most women were married; 46% housewife; 31.3% had no formal education and 27.9% had secondary or above Grade 12 | Associated with:  Lower educational status of partners; rural; frequent alcohol abuse by partner; early initiation of antenatal care; the age of women between 17–26 years; choice of partner by the women only |
| 9 | Babu et al., 2012  India | population-based  cross-sectional  1525 | unidirectional | physical: 7.1%  psychological:30.6%  sexual: 10.4%  Perpetrator(s):  husbands | During pregnancy | Most women were married; up to 50 years old; 79.7% housewife; 31.6% illiterate; 47.1% school education; 13.3% college and above | Associated with:  Urban living, higher maternal age and husbands' alcoholism  Women belonging to lower social groups were less likely to have physical DV  Factors such as higher prevalence of undesirable behaviours like denying adequate rest and diet, demand for more sex, not providing antenatal care and pressure for male child were also associated with DV in pregnancy. |
| 10 | Bahrami-Vazir et al., 2019  India | Population-based  Cross-sectional  525 | bidirectional | Women’s reported abuse of their husbands: 70%  (psychological aggression: 65%;  sexual coercion: 15%;  injury: 8%;  physical violence: 19%)  Women’s experience of abuse from their husbands: 67%  (psychological aggression: 58%;  sexual coercion: 30%;  injury: 16%;  physical violence: 22%) | during pregnancy (GW 24-30) | Most women were married between 1 and 15 years, living with their husband during the past 12 months, literacy of middle school level or more, and first formal marriage of both wife and husband; 92% housewives; received some form of education | Associated with:  Women's and husbands' satisfaction with their own occupations were predictors of both perpetration and victimization of IPA |
| 11 | Bernstein et al., 2016  South Africa | population-based  cross-sectional  623 | unidirectional | Overall IPV (12 months): 21%  emotional: 15%;  physical: 15%  sexual: 2% | during pregnancy (median GW 26) | Most women were HIV infected pregnant women initiating lifelong antiretroviral therapy; over 18 years old; (25% married, 41% living with partner) | Associated with:  Reported IPV was less likely among married women, and women who experienced IPV were more likely to score above threshold for substance use, depression and psychological distress. In addition, women who reported not discussing and/or not agreeing on pregnancy intentions with their partner prior to conception were significantly more likely to experience violence. |
| 12 | Bhatta & Assanangkornchai, 2019  Nepal | population-based  cross-sectional  165 for each timepoint  total: 660 | unidirectional | Overall DV: 26.2%  psychological: 15%  physical: 9.4%  sexual: 16.1%  6-10 weeks PP: 20.0%  psychological: 15.2%; physical 4.8%  sexual: 7.3% | during pregnancy and 6-10 weeks PP | Most women were married (58% arranged marriages); between 15-49 years old; 77% housewives; about 33% illiterate | Associated with:  Janjati ethnicity of the women, 2 to 5 years of married life compared with <2 years, the second and third trimesters of pregnancy compared with the first trimester, low education status of husbands, high controlling behavior of mothers-in-law, and previous history of domestic violence. |
| 13 | Carneiro et al., 2016  Brazil | population-based  cross-sectional  1026 | unidirectional | Overall IPV: 20.1%  Physical: 25.6% | During pregnancy (3rd trimester) | Most pregnant women were under 25 years old;  54% living with partner; 53.8% no own income;  have 62% less than 9 years of schooling | Associated with:  Tobacco smoking, alcohol consumption, and the use of illicit drugs, especially if throughout the  pregnancy, were associated with PVIP |
| 14 | Cervantes-Sanchez et al., 2016  Mexico | population-based  cross-sectional  102 | unidirectional | Overall: 18.6 %  physical: 10.8 %;  psychological: 5.9 %; sexual: 4% | during pregnancy | Most women were married; in third trimester; 39.2% workers; 35.3% have formal education |  |
| 15 | Charles & Perreira, 2007  USA | population-based cohort  longitudinal  2,310 | bidirectional | Against Mothers during Pregnancy  Overall: 8.5%  Physical: 1.7%  Emotional: 7.5%  Against Mothers 1 Year PP  Overall: 30%  Physical: 3.1%  Emotional: 17.3%  Coercion/Control: 21.4%  Against Partners during Pregnancy  Overall: 13.4%  Physical: 8.2%  Emotional: 7.0%  Against Partners 1 Year PP  Overall: 34.1%  Emotional: 13.3%  Coercion/Control: 27.7% | during pregnancy and 1 year PP | Most women were married; mean age mothers: 73% worked in year prior to birth; 33% less than high school; 20% college degree |  |
| 16 | Clark et al., 2009  Jordan | clinical-based  cross-sectional  390 | unidirectional | Overall DV: 15.4%  - Husband: 83.3%  - Others: 8.3%  - Both: 8.3% | during pregnancy |  | Associated with:  * women with less than secondary education  * Consanguineous relationship  * alcohol use by the husband  * acceptance of violence  * frequencies of quarreling  * less communication with natal family  *exposure to violence in childhood |
| 17 | Clarke et al., 2019  Uganda | clinical-based  cross-sectional  409 | unidirectional | Overall IPV: 26.7%  Any form of IPV: 70.6%  Sexual: 39.4%  Physical: 60.6%  Emotional: 59.6%  * women who experienced physical IPV were more likely to experience other forms  Lifetime IPV: 78.5% | during pregnancy |  | Associated with:  * acceptance of violence  * living with perpetrator  * partner’s daily alcohol use  * partner’s controlling behaviours  * Partner has relationship with other women while with her  * Partner has had a physical fight with another man  HIV test in last 12 months: 71.1%  currently HIV positive: 13%   - 48% married - 34% living with a man, not married |
| 18 | Das et al., 2013  India | population-based  cross-sectional  2,139 | unidirectional | Overall IPV: 15%  -Physical IPV: 12%  -Emotional IPV: 8%  -Sexual IPV: 2% | during pregnancy and 6 weeks PP |  | IPV was greater in:  *women living in poorer families  * Muslim families,  * women in paid employment  *women whose husbands used alcohol.  * reported a previous miscarriage |
| 19 | De Moraes et al., 2017  Brazil | population-based  cross-sectional  1,082 | bidirectional | Overall IPV: 30%  Physical  Minor (28.2%)  Severe (14%)  Against mothers 18.3%  - minor (17.5%)  -Severe (7.9%)  Against partners 25%  - minor (23.2%)  -severe (11.2%) | postpartum (up to 6 months after childbirth)  detailed estimated prevalence of IPV for subgroups available p.7 (table 4) |  | IPV was greater in:  * black adolescent mothers with less schooling and who were unemployed or underemployed  * child’s age  IPV act increased by women  * not living with a partner and who lived in households with more than one child less than 5 years of age  *with a history of inadequate or no prenatal care and fewer than 4 prenatal visits  *mothers not practicing exclusive breastfeeding and among those reporting difficulties in attending primary care clinics |
| 20 | Epuitai et al., 2019  Uganda | clinical-based  cross-sectional  180 | unidirectional | Overall IPV: 27.8%  physical 10.6%,  sexual 10.0%,  emotional 22.2%,  IPV before pregnancy: 55.6% | during pregnancy | Most women were married; between 15-24 years old; used contraceptive (44.9%); having unwanted pregnancy: (38.9%) | IPV was significantly associated with:  *household average monthly income  *IPV experience before pregnancy  *marital conflicts |
| 21 | Ezeanochie et al., 2011  Nigeria | clinical-based  cross-sectional  305 | unidirectional | Overall IPV: 32.5%  Psychological: 27.5%  Physical: 5.9%  Sexual: 9.8%%  Lifetime IPV (before HIV diagnosis): 30.3% | during pregnancy | Most women were diagnosed with HIV‐positive: before pregnancy (78.8%); having  HIV positive partners: 39.4%; having HIV‐positive child: 36.4% | IPV was significantly associated with:  *HIV‐positive multiparous pregnant women  *primary level of education  *history of IPV before HIV test  *those with an HIV‐positive child |
| 22 | Ezechi et al., 2009  Nigeria | clinical-based  cross-sectional  652 | unidirectional | Lifetime IPV: 65.7%  Before HIV diagnosis 25.9%  - increases after diagnosis 53.2%  Overall IPV after HIV diagnosis 74.1%  Verbal: 51.7%  economic deprivation: 15.7%  physical 8.1%  sexual deprivation: 21.6% | during pregnancy | Most women were aged between 20 -39; having  HIV negative spouses (55.3%) | IPV was significantly associated with:  *Low socioeconomic class  *negative HIV status of women’s spouse  *diagnosis of HIV |
| 23 | Farrokh-Eslamlou et al., 2014  Iran | clinical-based  cross-sectional  313 | unidirectional | Lifetime IPV (60.1%)  Physical/emotional violence entire marriage life (46.9%)  Overall IPV (55.9%)  Types of IPV:  psychological (43.5%),  physical (10.2%)  sexual (17.3%) | during pregnancy  first 48 h postpartum | Most women were married <4years (53.4%); >5 years(46.7%); and primi-gravida (66.5%) | IPV was significantly associated with:  *lower education & unemployment of the husbands  *marriage duration of 5–9 years  * gravidity of two |
| 24 | Fawole et al., 2008  Nigeria | clinical-based  cross-sectional  534 | unidirectional | IPV before pregnancy (12 months): 41.5%  Overall IPV during pregnancy 17.7%  Verbal abuse (66.2%)  Other forms: included flogging (10.8%), slaps (9.5%), threats of violence (6.8%) and forced sexual intercourse (2.7%). | before and during pregnancy |  | DV significantly associated with:  *Women aged 20 years or less  *Women with less than secondary level of education  *Alcohol intake in the partner  * IPV before pregnancy (12 months) |
| 25 | Fekaduet al., 2018  Ethiopia | clinical -based  cross-sectional  450 | unidirectional | Overall DV= 58.7%  emotional = 57.8% physical= 32.2%  sexual =7.6% | during pregnancy | DV was significantly associated with:  * unemployed women with no salary of their own  * husbands used alcohol daily  * believing women’s responsibility to be pregnant and obey their partners |  |
| 26 | Field et al., 2018  South Africa | clinical-based  cross-sectional  (mixed methods) 376 | unidirectional | Overall IPV: 15%  emotional/verbal: 81%  physical: 76%  sexual: 26%  multiple forms of abuse: 46%  Lifetime abuse 50% | during pregnancy | IPV was significantly associated with women:  * younger than 29  * food insecure  * stable relationship but not married  *not pleased with pregnancy  Unemployed status: 58%  Food insecurity = 62%  Not pleased with pregnanc40%  Major Depressive Episode: 40%  Any anxiety disorder = 36%  Suicidal Behaviour: 31%  Alcohol and other drug use:20%  History of mental health problems: 24% |  |
| 27 | Finnbogadóttir et al., 2014  Sweden | clinical-based  cross-sectional  (first part of longitudinal cohort study)    1,939 | unidirectional | lifetime DV: 39.5%  emotional 19.5% (66.3% by male)  physical 29.3% (74.2% by male)  sexual 15.7% (37.1% by male)  during pregnancy:  overall DV: (1.5%)  emotional 1.0%  physical 0.4%  sexual 0.1% | during pregnancy |  | IPV was significantly associated with:  *history of violence  *employment- and financial distress  *smoking and snuffing  *unintended pregnancies  *history of miscarriages and abortion  *single or living apart  *lack of sleep  *presence of several symptoms of depression  *inability to use their own resources to maintain and improve their health in stressful situations |
| 28 | Finnbogadóttir et al., 2016  Sweden | clinical-based    longitudinal    1,939 | unidirectional | Lifetime DV: 44.3 %  during pregnancy:  Overall DV: 2%  Emotional (1.6%), Physical (0.7%),  Sexual (0.1%) | during pregnancy (early/late) |  | IPV was significantly associated with:  *history of violence  *low educational status  *living Single/living apart  *financial distress  *unintended pregnancy  *lack of sleep  *presence of several symptoms of depression  *inability to use their own resources to maintain and improve their health in stressful situations |
| 29 | Fiorotti et al., 2018  Brazil | clinical-based  cross-sectional  302 | unidirectional | Lifetime DV (12 months): 43%  physical 7.6%  during current pregnancy:  physical 4.6% | during pregnancy |  | DVwas significantly associated with:  *aged between 31-43 years old *having three or more pregnancies *evangelical religion (Catholics)  *history of abortion  *does not currently have a partner |
| 30 | Fisher et al., 2003  Israel | clinical-based  cross-sectional  270 | unidirectional | Overall physical DV 5.4%  Psychological (21.6%)  physical (minor 20.3% and sever 8.1%)  sexual (4.1%). | during pregnancy |  | DV was significantly associated with:  *socioeconomic status, work status, and degree of religiosity  *low educational status of partners  *low income  *partner's unemployment  *Ethnicity (Jewish women of Sephardic descent or Muslim women)  *religious women/men |
| 31 | Flanagan et al., 2014  USA | clinical-based  longitudinal  baseline 180  (follow-up with 122) | bidirectional | Overall Sexual IPV (Baseline>Follow-up)  Against women 11.7% > 12.3%  Against partners 9.4% > 7.4%  IPV types at baseline:  Against women  -Sexual only 1.7%  -Sexual+ psychological/ physical 10%  Against partners  -Sexual+ psychological/ physical 9.4%  At follow-up:  Against women  -Sexual only 1.6%  -Sexual+ psychological/ physical10.7%  Against partners  -Sexual only 0.8%  -Sexual+ psychological/ physical 6.6% | during pregnancy and 6 wks postpartum | Most women were first 18 weeks of pregnancy; at least 18 years; in contact with either their intimate partner or child's father |  |
| 32 | Garg et al., 2019  India | clinical-based  cross‑sectional  1,500 | unidirectional | Overall DV 29.7%  physical 26.9%  economical, 37.0%  emotional/verbal 79.1%  sexual 33.2% | during pregnancy |  | DVwas significantly associated with:  *belonging to general caste  *subjects of Hindu religion  *illiteracy  **spouses were unemployed |
| 33 | Gartland et al., 2011  Australia | clinical-based  longitudinal  1,301 | unidirectional | Overall IPV (12 month)17%  physical 2.2%  emotional 9.0%  both 5.4% | during pregnancy and postpartum (3, 6, 12 months) |  | IPV was significantly associated with:  *maternal age, relationship status, income and education level. |
| 34 | Groves et al., 2015  South Africa | clinical-based  longitudinal  445 | unidirectional | Overall IPV 42.25 %  IPV during pregnancy: 21.35 %  psychological 16.63 %  physical 8.76 %  sexual 3.15 %  IPV at 4 months post. 24.94 %  IPV at 4-9 months post. 17.75 % | during pregnancy and postpartum (first 9 months): |  | In both periods, IPV was significantly associated with:  *Age (older age at lower risk)  *history of IPV in the current relationship  HIV-positive at baseline: 35.51%  Pregnancy unintended: 79.73% |
| 35 | Gyuse et al., 2009  Nigeria | clinical-based  cross-sectional  340 | unidirectional | Lifetime DV 63.2%  Overall DV in current pregnancy: 12.6%  physical 26.5%  verbal 38.0%  sexual 10.7%  emotional 1.4%  Both physical & exual 7%  Both physical & verbal 14% | during pregnancy |  | IPV was significantly associated with:  *history of previous abuse  *occupation (Self-employed women were the most abused followed by full-time housewives and civil servants) |
| 36 | Habib et al., 2018  Pakistan | clinical-based  cross-sectional  1,000 | unidirectional | Overall DV 35%  physical minor 27%  physical severe 6% | during pregnancy |  | IPV was significantly associated with:  * residents of urban areas  * older age, being uneducated & belonging to poor socioeconomic status |
| 37 | Haron et al., 2018  Malaysia | clinical-based  cross-sectional  1,200 | unidirectional | Overall DV 35.9%  emotional (29.8%)  physical (12.9%)  sexual (9.8%) | during pregnancy |  | DV was significantly associated with:  *low education  *unmarried status (for emotional & sexual)  *drug use (emotional & physical)  *violence during childhood (emotional)  *multipara women (sexual)  *inadequate ante-natal care  * women’s attitude towards gender norms, justifying violence, refuse sex (emotional / sexual) |
| 38 | Harrykissoon et al., 2002  USA | population-based  prospective, longitudinal  570 (analytical sample, at least 4 out of 5 follow-ups had to be completed),  770 (total sample size) | unidirectional | No overall IPV was provided  at 3 months pp: 21.3%  at 6 months pp: 16.1%  at 12 months pp: 17.7%  at 18 months pp: 17.7%  at 24 months pp: 12.8% | IPV assessed within 48 hours of delivery (including IPV during preceding 12 months), at 3, 6 (recalling former 3 months), 12, 18, and 24 months (recalling former 6 months) pp | analytical sample  limited to adolescents (18 years or younger), M = 16.8 years (SD = 1.2)  ethnicity: 38% Mexican American, 32% African American, 30% European American  marital status: 35% married (for those experiencing IPV, values on current relationship provided for pp assessments)  education: 15% graduated from high school or obtained a graduate equivalency diploma  occupation: 14% employed (referring to last 3 months of pregnancy)  15% already had child(ren) |  |
| 39 | Hellmuth et al., 2013  USA | clinic-based  prospective, longitudinal  180  122 at follow-up | bi-directional | baseline perpetration:12%  follow up perpetration: 7% (no victimization)  Women’s perpetration:  overall IPV:  baseline: 72.2%  follow-up: 64.8%  psychological minor: 21.1%, 20.5%  physical severe: 9.4%, 12.3%  Women’s victimization  physical minor: 67.7%, 54.1%  physical severe: 8.3%, 4.1%  psychological minor: 13.3%, 10.7% | IPV during first 18 weeks of pregnancy (referring to time since pregnancy began), at 6 weeks postpartum (referring to time since baseline) | “population of women of low socioeconomic status and childbearing age” (p. 2)  age: at least 18 years  ethnicity: 79% Caucasian  religion: 81% Christian  household income: 87% with less than $50.000 yearly  education: 80% high school  marital status: 23% married, 64% partnered  4% divorced, 7% not in relationship  higher psychological and minor physical IPV perpetration at baseline was associated with:   - higher IPV victimization, - reported partner alcohol misuse, - stress, depression, and lower dyadic adjustment   higher sever physical IPV perpetration at baseline was associated with:   - lower dyadic adjustment | CAVE: values provided in abstract differ from values given in tables  authors indicated use of three subscales (psychological, minor physical, and severe physical) but not all corresponding values seem to be provided (see table 1) |
| 40 | Islam et al., 2018  Bangladesh | population-based  cross-sectional  426 | unidirectional | during pregnancy  overall IPV: 66.4%  physical violence: 35.2%  sexual violence: 18.5%  psychological violence: 65.0%  after pregnancy:  overall IPV: 63.6%  physical violence: 32.2%  sexual violence: 15.5%  psychological violence: 60.8% | before (12 months prior to pregnancy), during and after (first 6 months postpartum) pregnancy | participants were “new mothers” (p. 1) in first 6 months postpartum  age: 15-24 (54.5%)  25-44 (39.4%)  35 and older (6.1%)  marital status: 100% married  education: “relatively low level of literacy” (p. 7); 8.2% without formal education, 24.4% primary education, 67.4% secondary and higher education  religion: 93.2% Muslim  family monthly income: 38.3% with less than 8.501 BDT ($109)  59.9% had more than one child | before pregnancy:  overall IPV: 71.8%  physical violence: 52.8%  sexual violence: 21.1%  psychological violence: 67.4% |
| 41 | Islam et al., 2017  Bangladesh | population-based  cross-sectional  426 | unidirectional | IPV during pregnancy:  66,4%  -> physical: 35.2 %  -> psychological: 65%  -> sexual: 18.5 % | during pregnancy | married women, living with their husband for at least 2 years  age between 15 and 49 years (52% were between 15 and 24 years old)  first six months postpartum  27,7 % of intimate partner had controlling behaviour  63,4 % of the marriages involved dowry demands  more than half of the women experienced parental violence or violence before the age of 15  associated factors:  – psychological IPV often occurs in isolation  – all forms of IPV significantly more prevalent among women with no formal education and lower family incomes  – women living in nuclear families were at higher risk for psychological violence than women living in extended families  – higher levels of IPV in women living in rural areas  – higher risk for all forms of IPV for women whose husband had no formal education and is drug user  – mothers of three or more children were more likely to be physically and sexually victimized  – women who reported a dowry demand, low decision-making autonomy, higher acceptance of traditional gender roles, low self-esteem, low social support during pregnancy were at higher risk for physical and psychological IPV  – unintended pregnancy is risk factor  – husband with moderate to high controlling behaviour as a risk factor (10 times more likely) |  |
| 42 | Jahanfan & Malekzadegan, 2007  Iran | population-based  cross-sectional  1,800 (97%) | unidirectional | overall DV: 60.6%  physical DV: 14.6% (5.3% severe, 55.5% moderate, 39.2% mild)  psychological DV: 60.5% (24.6% severe, 8.5% moderate, 66.9% mild)  sexual DV: 23.5% (3.4% severe, 13.4% moderate, 83.2% mild) | during pregnancy and PP | age: M = 25.80 (SD = 5.27), range 15-45  education (some illiteracy): 51.3% had “primary certificate” (p. 645)  women's income: M = 79.14 (SD = 43.03) Toman  family income: M = 112.76 (SD = 71.28)  37% primiparous | data was extracted from abstract. Full text unavailable. |
| 43 | Jain et al., 2017  India | clinic-based  cross-sectional  400 | unidirectional | IPV during pregnancy: 12.3%  emotional IPV: 10.7%  physical: 10%  sexual: 1.8% | during pregnancy (20 – 28 GW) | pregnant women attending outpatient clinic  – most of them had school education  – mostly urban background  – almost 100% housewives  – most of them had a middle socioeconomic status  – most of them lived in an extended family  risk factors:  –husband’s desire for a son  – intimate partner older than the woman  – longer duration of marriage  – multigravidity  – lower socioeconomic status |  |
| 44 | Jamshidimanesh et al., 2013  Iran | population-based  cross-sectional  600 | unidirectional | IPV during pregnancy: 56.3%  emotional IPV postpartum : 51.3%  physical IPV postpartum: 5%  sexual: no mentioning  IPV before pregnancy: 17% | during pregnancy and PP | risk factors:  – premature labor rate was higher amongst abused women  – employment status: higher employment rate in abused women  – method of delivery: less vaginal delivery in abused women  – lower education level of husband | Lack of information on sexual abuse; pretest showed that women don’t experience sexual abuse and they therefore left out a question regarding sexual violence  no differentiated numbers on which form of violence is experienced during pregnancy  the study was carried out several hours after giving birth |
| 45 | Jeremiah et al., 2011  Nigeria | population-based  cross-sectional  500 | unidirectional | IPV during pregnancy: 7.8 %  verbal IPV: 43.5%  physical: 11.2%  sexual: 1.8%  economic violence: 6.8% | during pregnancy (second half) | patients between 18 and 49 years old, 98% were married  risk factors:  – low education  – low parity  – younger women  – substance abusing husbands  – unemployment both in women and intimate partners |  |
| 46 | Johnson et al., 2003  England | clinic-based  cross-sectional  475 (95%) | unidirectional | overall IPV: 17%  physical violence: 14.7%  emotional violence: 14.3% | during pregnancy | Abuse was highest in the group of 26-3- years.  It was more prevalent in single women. |  |
| 47 | Karaoglu et al., 2006  Turkey | population-based  cross-sectional  824 (91.6)  580 in urban areas  244 in rural settlements | unidirectional | during pregnancy:  overall IPV: 31.7%  physical violence: 8.1%  emotional violence: 26.7%  sexual violence: 9.7%  lifetime:  overall IPV: 36.3%  physical violence: 16.3%  emotional violence: 30.8%  sexual violence: 8.5.7% | during pregnancy | average age: 26 ± 0.2 years  education: 10.2%  illiterate 55.1% completed 5 years of primary school education  frequency of illiterate partners: 2.5% (significantly lower than women) | When those who were exposed to overall violence during pregnancy were examined separately, it was observed that the frequency of physical violence was decreased while the frequencies of emotional and sexual violence were  increased (p <0.05) |
| 48 | Karmaliani et al., 2008  Pakistan | population-based  cross-sectional  1,368 | unidirectional | during pregnancy:  overall violence: 38%  verbal abuse: 24%  physical and/or sexual abuse: 14%  6 months prior to pregnancy:  overall violence: 47%  verbal abuse: 16%  physical and/or sexual abuse: 16% | during pregnancy and 6 months prior to pregnancy | average age: 27 years  first sexual intercourse less than 18 years: 28%  previous pregnancy: 82%  polygamy: 4% |  |
| 49 | Kaye et al., 2002  Uganda | clinic-based  cross-sectional  379 | unidirectional | overall moderate-to-sever domestic violence: 57% | during pregnancy | IPV was significantly associated with:  exposure to childhood violence, witnessing IPV as a child, polygamy, first pregnancy, and adolescent pregnancy | data extracted from abstract alone |
| 50 | Khaironisak et al., 2017  Malaysia | clinic-based  cross-sectional  1,200 | unidirectional | overall IPV: 35.9%  psychological violence: 29.8%  physical violence: 12.9%  sexual violence: 9.8% | during pregnancy | IPV was significantly associated with: women’s use of drugs, having had exposure to childhood violence, violence supporting attitude, two or more children, and having partners who were smokers, alcohol drinkers, and had controlling behavior. | data extracted from abstract alone |
| 51 | Kita et al., 2014  Japan | -  cross-sectional  302 | unidirectional | overall IPV: 15.9% | during pregnancy | IPV was significantly associated with:  age over 30, multipara, previous abortion experience, and male partner aged under 30. | data extracted from abstract alone |
| 52 | Koenig et al., 2002  USA | clinic-based  cross-sectional  HIV infected: 336  HIV-uninfected: 298 | unidirectional | overall IPV: 8.9% | during pregnancy | HIV infected and uninfected.  Disclosure-related IPV occurred but was rare. | seropositivity was reported to be not positively associated with higher IPV victimization |
| 53 | Kothari et al., 2015  England | population-based  longitudinal (19-22 years)  women with maternal lifetime eating disorder (ED): 174  women with or without pregnancy shape and weight concerns and/or purging behavior: 189  women with no ED: 8,723 | unidirectional | IPV for women with lifetime ED during or after perinatal period:  physical violence: 9.6% / 14.3%  emotional violence: 24% / 28% | during and after perinatal period | three groups of women were investigated:   - women with lifetime ED - women with or without pregnancy shape and weight concerns and/or purging behavior - women with no ED   women with lifetime ED and pregnancy shape and weight concerns and/or purging had higher odds of reporting physical IPV at 8-33 months after delivery. | lifetime ED was associated with higher IPV during perinatal period |
| 54 | Lukasse et al., 2014  Belgium, Iceland, Denmark, Estonia, Norway, and Sweden | clinic-based  prospective, longitudinal  7,174 | unidirectional | sexual abuse: 0.4%  physical abuse: 2.2%  emotional abuse: 2.7% | during pregnancy | significant differences between countries in our sample were observed:   - nearly a quarter of the women were below 25 years of age in Estonia, but only around 3% in Denmark. - Norway had the highest proportion of educated women (13 years or more of education), while the lowest proportion was found in Estonia. - Most women were married or cohabiting. - Iceland and Estonia had the highest proportion of women not married or cohabiting, as well as the highest proportion of women who were unemployed or on social benefit. | Current moderate or severe suffering from reported emotional abuse was highest among Icelandic women (88.8%) and lowest among Estonian women (68.1%). |
| 55 | Lutgendorf et al., 2009  USA | clinic-based  cross-sectional  1,104 (95%) | unidirectional | domestic violence: 14.5%  sexual violence: 0.9% | during pregnancy | women were in the military.  The majority of participants were in their 20s, white, and high school graduates with some college. Most of our participants were dependent spouses of Navy personnel, and the majority were enlisted, rank E6 (mid enlisted) and below. |  |
| 56 | Mahenge et al., 2016  Tanzania | population-based  cross-sectional  500  (97%) | unidirectional | IPV (sexual and/or physical) during pregnancy: 18.8%  physical violence during pregnancy: 12.4%  sexual violence during pregnancy: 9%  psychological violence during pregnancy: 31%  economic violence during pregnancy: 15%  controlling behaviour during pregnancy: 48.4%  IPV (sexual and/or physical) postpartum: 8.2 %  physical IPV postpartum: 5.2 %  sexual IPV postpartum: 3.8%  psychological IPV postpartum: 17.8%  economic IPV postpartum: 11.4 %  controlling behaviour IPV postpartum: 44,4%  IPV during pregnancy AND postpartum: 4,8 %  psychological violence / controlling behaviour during pregnancy AND postpartum: 12,4 % | during pregnancy until 9 months PP | women attending clinics for children under the age of 5  all attending mothers aged 18 years and older, physically fit without cognitive impairment were invited to participate |  |
| 57 | Makayoto et al., 2013  Kenya | population-based  cross-sectional  300 | unidirectional | IPV during pregnancy (at least one form): 37%  psychological violence during pregnancy: 29%  sexual violence during pregnancy: 12%  physical violence during pregnancy: 10% | 12 months prior to and during pregnancy | pregnant women, mean age of 24 years, majority were married, majority had complete primary school education, most of the partners did not consume alcohol, 285 participants had been tested of HIV (18% were HIV positive), some of them lived in polygamous relationships (one male, several female) |  |
| 58 | Malan et al., 2018  South Africa | population-based  cross-sectional  150 | unidirectional | 12- months IPV rates:  emotional IPV/controlling behaviour: 32%  physical violence: 29%  sexual violence: 20% | during pregnancy | pregnant women attending antenatal care, 18 years or older | 12 month IPV rates were checked -> IPV might have happened prior to pregnancy -> how do we deal with that? |
| 59 | Marcacine et al., 2013  Brazil | population-based  cross-sectional  207 | unidirectional | IPV during pregnancy:  - 36,7%  - psychological: 32,9%  - physical: 14%  - sexual: 0,1%  IPV postpartum:  - 25,6%  - psychological: 25,1%  - physical: 4,3%  - sexual: 0,5% | during pregnancy and PP | majority of women were young, without vices, Catholics, with high school education, living with a partner who is family provider, average time of 7 years relationship, almost half of the women were unemployed or housewife, almost half of them resided in their own property, family income was between one and three minimum Brazilian wages  partners of the studied group was mostly young people, good education, workers, non-users of drugs |  |
| 60 | Massumi Okada et al., 2015  Brazil | population-based  cross-sectional  385 | unidirectional | IPV during pregnancy: 34,6% | during pregnancy | mostly young women, married. with 9 to 11 years of education, Catholic, residents in their own home, without a paid job, partner was main family provider |  |
| 61 | Modiba et al., 2011  South Africa | population-based  cross-sectional  163 | unidirectional | IPV during pregnancy: 41%  -physical: 17%  -emotional: 26%  -sexual: 5%  - both physical and emotional: 9% | during pregnancy | pregnant women between 18 and 41 years, including single / engaged / married / divorced women, majority had completed senior school, mostly black South Africans, Christians |  |
| 62 | Mohammad-Alizadeh-Charandabi et al., 2016  Iran | clinic-based  comparative cross-sectional  136 (aged between 15 to 19)  272 (aged between 20-29)  408 in total | bidirectional | women’s perpetration:  15 to 19 year olds:  72,1%  20 to 29 year olds:  71,0%  women’s victimization:  15 to 19 year olds:  69%  20 to 29 year olds:  62 % | during pregnancy (24-30 GW) | education level of adolescents significantly lower than that of the adults, rate of housewives higher among adolescents |  |
| 63 | Mohammadhosseini et al., 2010  Iran | population-based  cross-sectional  300 | unidirectional | IPV before pregnancy:  emotional: 41.7 %  physical: 16.7 %  sexual: 21 %  any type: 51.7 %  IPV during pregnancy:  emotional: 33%  physical: 10%  sexual: 17.3%  any type: 42 %  IPV postpartum:  emotional: 42.7%  physical: 14.7 %  sexual: 25 %  any type: 53.5 %  In all periods:  emotional: 53,5%  physical: 26,7 %  sexual: 34,7 %  any type: 64,7 % | during pregnancy and PP (6 to 18 months) | married women (18 – 40 years) with a child between 6 and 18 months, most women had one child  risk factor:  – abuse before pregnancy and during pregnancy  – experience of abuse of other women (children of abused mothers are likely to become abused wifes)  – lower education in husband  – living in extended family, lower education in women, economic dependency, women’s age marriage below 18 years = predictor for physical abused  – opium use by husband, husband’s smoking, =predictors for emotional abuse  “causes” for abuse  – poor relationship with husband’s family  – discipline of the children  – financial problems |  |
| 64 | Muzrif et al., 2018  Sri Lanka | population-based  cross-sectional  2,088 | unidirectional | lifetime IPV:  38,6 %  IPV during pregnancy:  15,9% | during pregnancy (6-40 GW) | – participants were recruited both in a rural area (tea plantation) and urban area  participants from capital district:  16 to 44 years of age, mostly older than 31, mostly higher education, mostly employed, 99,5% married, partner’s education mostly high, partners mostly employed, mostly low income  ethnicity:  Sinhala: 73,6%  Tamil: 12,6 %  Muslim: 12,9 %  Others/missing: 0,8%  participants from tea plantation area:  16 to 44 years of age, mostly younger than 31, mostly low education, 53% unemployed, 99% married, partner’s education mostly low, partners mostly employed, mostly low income  ethnicity:  Sinhala: 2,5%  Tamil: 96,1 %  Muslim: 1,4 %  Others/missing: 0,0%  risk factor:  – women living in rural area (tea plantation sector) were more likely to be “ever abused” and “currently abused”  – “ever abused” was associated with living in the tea plantation sector, being employed, living far away from gender-based violation care center, being of Muslim ethnicity |  |
| 65 | Nguyen et al., 2018  Vietnam | population-based  cross-sectional  1,309 | unidirectional | IPV during pregnancy: 35.2%  Emotional violence:  32.2%  physical violence: 3.5% sexual violence: 9.9% | during pregnancy ( 30th–34 GW) | mean age 27 years, 44% had high school education or higher, employment rate 33%,  risk factors:  – previous experience of IPV (before pregnancy)  – lack of social support  – younger age |  |
| 66 | Okour & Badarneh, 2011  Jordan | population-based  cross-sectional  303 | unidirectional | IPV during pregnancy: 40.9%  Emotional violence:  28.1%  physical violence: 34.7%  sexual violence: 15.5% | during pregnancy | mean age 28,3 years, some women had up to 14 pregnancies,  risk factors:  – living in urban area,  – unplanned pregnancy  – six or more pregnancies including the current one  – four or more female children  – pressure to have a male child |  |
| 67 | Olagbuji et al., 2010  Nigeria | clinic-based  cross-sectional  502 | unidirectional | before, during, and after pregnancy: 43.8%  before pregnancy: 43.3%  during pregnancy: 28.3%  after pregnancy (puerperium^^[[1]](#footnote-1)^^): 0.8% | before, during, and after pregnancy (puerperium) | Violence in pregnancy and the puerperium was more common:   - in HIV- positive women - who reported experiencing violence within the 12 months before the pregnancy - who reported regular alcohol consumption during pregnancy and the puerperium | Psychological violence was the commonest form of violence in the 12 months before the pregnancy, in all the trimesters of pregnancy, and in the puerperium.  More participants reported experience of physical  violence in the first trimester than in the second and third trimesters of pregnancy.  Sexual violence was more common in the third trimester compared with the first and second trimester. All forms of violence were least common in the puerperium. |
| 68 | Onoh et al., 2013  Nigeria | clinic-based  cross-sectional  321 (91.7%) | unidirectional | overall IPV: 44.6%  verbal abuse: 60.1% | during pregnancy | IPV was most common with:  women <20 years of age  duration of marriage between 6 and 9 years  polygamy  being christian (pentecostals), moslem and atheist. | 7.7% were hospitalized as result to physical and emotional abuse |
| 69 | Owaka et al., 2017  Kenya | clinic-based  cross-sectional  224 (94.1%) | unidirectional | overall IPV: 66.9%  physical violence: 29.9%  psychological violence: 55.8%  sexual violence: 39.2% | during pregnancy | overall IPV was significantly associated with:   - alcohol intake by partners - partner’s level of education   psychological and sexual violence was significantly associated with:   - age of partner (less than 25 years) and pregnant women |  |
| 70 | Oweis et al., 2010  Jordan | clinic-based  cross-sectional  316 | unidirectional | physical violence: 10.4%  emotional violence: 23.4%  verbal violence: 23.7%  sexual violence: 5.7% | during pregnancy | overall IPV was significantly associated with:   - unplanned pregnancy - the pregnant women’s perception of their husband’s violent attributes - the women’s low self- esteem |  |
| 71 | Peedicayil et al., 2004  India | population-based  cross-sectional  9,938 | unidirectional | physical prevalence of moderate to severe violence: 13% | during pregnancy | overall IPV was significantly associated with:   - illiteracy and employment of women - dowry harassment after marriage - absent or poor social support - witnessing IPV as a child - exposure to childhood violence - husband being drunk regularly - husband having an affair - husband accusing wife of having extramarital affair - low education of husband - having more than 3 children - household crowding | lifetime physical violence: 41% |
| 72 | Pitter & Dunn, 2018  Jamaica | clinic-based  cross-sectional  185 | unidirectional | overall IPV: 41% | during pregnancy | at risk:  23-29 years old, single (66.6%), employed women (44%) with primary education (66.6%) and had unplanned pregnancy (65%) | those who sought help: 11%  data extracted from abstract; no full text found |
| 73 | Romero-Gutierrez et al., 2011  Mexico | clinic-based  cross-sectional  1,623 | unidirectional | overall violence: 43.8%  psychological violence: 72.9%  physical violence: 15.8%  sexual violence: 11.3% | during pregnancy | IPV was significantly associated with:   - monthly family income - childhood violence for women and men |  |
| 74 | Rubertsson et al., 2010  Sweden | clinic-based  longitudinal  2,563 | unidirectional | overall IPV: 2% | first year PP | average age: 29 years  primiparous women: 44%  multiparous women: 56% |  |
| 75 | Salari & Nakhaee, 2008  Iran | clinic-based  cross-sectional  416 (90%) | unidirectional | emotional violence: 35%  physical violence: 25% | during 48 after delivery | The mean age (±SD): 28.0 ±5.6  all were married.  urban residents: 89.2%  multiparous women: 78.8%  unplanned pregnancy: nearly 16%  unplanned pregnancies and multiparity were significantly associated with IPV |  |
| 76 | Shamu et al., 2013  Zimbabwe | clinic-based  cross-sectional  2,042 | unidirectional | overall IPV: 63.1%  physical and/or sexual violence: 46.2%  sexual violence: 38%  physical violence: 15.9%  severe violence: 10% | during pregnancy | overall IPV was associated with:   - having a younger malepartner - gender inequities - past abuse - problem drinking - partner control of woman’s reproductive health - risky sexual practices. - HIV status was not associated with either IPV or severe IPV, but reporting a partner with a known HIV status was associated with a lower likelihood of severe abuse. |  |
| 77 | Sherstha et al., 2016  Nepal | clinic-based  cross-sectional  404 | unidirectional | overall DV: 27.2%  sexual violence: 17.3%  psychological violence: 16.6%  physical violence: 3% | during pregnancy | overall DV was associated with:   - husbands’ characteristics such as age (younger than 25), education (illiterate), alcohol consumption, extramarital relationship, and controlling behavior of the husband. - support from the friends, being a member of any community group or organization and women’s attitude towards wife beating. | no subgroup analysis |
| 78 | Silverman et al., 2016  India | clinic-based  cross-sectional  1,061 | unidirectional | overall IPV: 28.4% | during pregnancy and/or PP | age: 17 to 45 years  (55.7%) under age 25 years | 2.6% reported perinatal violence from in-laws,  and 49% reported one or more forms of perinatal gender-based household maltreatment (GBHM) |
| 79 | Sohail & Qadir, 2009  Pakistan | clinic-based  cross-sectional  543 | unidirectional | overall DV: 5.7% | during pregnancy | - | data extracted from abstract |
| 80 | Spangenberg et al., 2016  Ghana | clinic-based  cross-sectional  153 | unidirectional | overall IPV: 46%  emotional violence: 34%  physical abuse: 17%  sexual abuse: 15% | during perinatal period | mean age of women: 28 years  urban areas: 55%  employed: 66% |  |
| 81 | Stöckl et al., 2010  Tanzania | clinic-based  cross-sectional  2,503 | unidirectional | physical violence: 19% | during pregnancy | violence started first during pregnancy  data was extracted from abstract | IPV was significantly associated with:   - being unmarried - having had children from different fathers - partner’s unfaithfulness - partner’s refusal to use contraception |
| 82 | Umoh et al., 2012  Nigeria | clinic-based  cross-sectional  442 (88.4%) | unidirectional | physical violence: 10.3% | current and past pregnancies |  | IPV was significantly associated with:   - number of deliveries/partiy - being married - husband’s occupation |
| 83 | Valladares et al., 2005  Nicaragua | population-based  cross-sectional  478 | unidirectional | overall IPV: 32%  emotional abuse: 32%  physical abuse: 13%  sexual abuse: 7% | during pregnancy | Violence reported commonly by young women. Abused women reported pregnancy as unwanted more frequently and late booking at antenatal care | IPV was significantly associated with:   - lifetime IPV |
| 84 | Van Parys et al., 2014  Belgium | clinic-based  cross-sectional  1,894 | unidirectional | overall IPV: 10.6%  sexual violence: 10.1%  physical violence: 0.5% | during pregnancy |  | IPV was significantly associated with:   - divorced/single - no primary or secondary education |
| 85 | Yang et al., 2006  Taiwan | clinic-based  cross-sectional  1,143 | unidirectional | physical violence: 6.9% | during pregnancy |  | IPV was significantly associated with:   - had fewer years of education - unemployed husbands - with a patriarchal family situation - husbands who had alcohol, cigarette and non-prescription drug use |
| 86 | Yohannes et al., 2019  Ethiopia | clinic-based  cross-sectional  299 | unidirectional | physical violence: 44.2%  psychological violence: 39.1%  sexual violence: 23.7% | during pregnancy |  | IPV was significantly associated with:   - illiterate women - husband’s alcohol consumption, - husband’s occupation (farmer) - husband’s history of arrest |

Appendix A. Search strategy for PubMed

Appendix B. Extraction table for included studies

1. the period of about six weeks after childbirth during which the mother's reproductive organs return to their original non-pregnant condition. [↑](#footnote-ref-1)
